# Simple Process-Based Simulators for Generating Spatial Patterns of Habitat Loss and Fragmentation: A Review and Introduction to the *G-RaFFe* Model

**DOI:** 10.1371/journal.pone.0064968

**Published:** 2013-05-28

**Authors:** Guy Pe'er, Gustavo A. Zurita, Lucia Schober, Maria I. Bellocq, Maximilian Strer, Michael Müller, Sandro Pütz

**Affiliations:** 1 UFZ - Helmholtz Centre for Environmental Research, Department of Conservation Biology, Leipzig, Germany; 2 UFZ - Helmholtz Centre for Environmental Research, Department of Ecological Modelling, Leipzig, Germany; 3 CONICET, Instituto de Biología Subtropical, Facultad de Ciencias Forestales, Universidad Nacional de Misiones. Puerto Iguazú, Misiones, Argentina; 4 CONICET, Departamento de Ecología, Genética y Evolución, FCEN, Universidad de Buenos Aires, Buenos Aires, Argentina; 5 UFZ - Helmholtz Centre for Environmental Research, Department of Bioenergy, Leipzig, Germany; National Institute of Water & Atmospheric Research, New Zealand

## Abstract

Landscape simulators are widely applied in landscape ecology for generating landscape patterns. These models can be divided into two categories: pattern-based models that generate spatial patterns irrespective of the processes that shape them, and process-based models that attempt to generate patterns based on the processes that shape them. The latter often tend toward complexity in an attempt to obtain high predictive precision, but are rarely used for generic or theoretical purposes. Here we show that a simple process-based simulator can generate a variety of spatial patterns including realistic ones, typifying landscapes fragmented by anthropogenic activities. The model “*G-RaFFe*” generates roads and fields to reproduce the processes in which forests are converted into arable lands. For a selected level of habitat cover, three factors dominate its outcomes: the number of roads (accessibility), maximum field size (accounting for land ownership patterns), and maximum field disconnection (which enables field to be detached from roads). We compared the performance of *G-RaFFe* to three other models: *Simmap* (neutral model), *Qrule* (fractal-based) and *Dinamica EGO* (with 4 model versions differing in complexity). A PCA-based analysis indicated *G-RaFFe* and *Dinamica* version 4 (most complex) to perform best in matching realistic spatial patterns, but an alternative analysis which considers model variability identified *G-RaFFe* and *Qrule* as performing best. We also found model performance to be affected by habitat cover and the actual land-uses, the latter reflecting on land ownership patterns. We suggest that simple process-based generators such as *G-RaFFe* can be used to generate spatial patterns as templates for theoretical analyses, as well as for gaining better understanding of the relation between spatial processes and patterns. We suggest caution in applying neutral or fractal-based approaches, since spatial patterns that typify anthropogenic landscapes are often non-fractal in nature.

## Introduction

Landscape simulators are widely applied in landscape ecology for generating virtual landscapes differing in structure and composition [1]–[4]. Especially when combined with population dynamics models, these landscapes serve as templates for analyzing dispersal, connectivity, population dynamics, and community processes in fragmented, patchy or heterogeneous landscapes [5]–[7]. The power of such models lies in their flexibility and their capacity to control for landscape structure and composition in order to separate between attributes such as habitat loss and fragmentation, that in reality are often strongly interrelated [8], [9].

We differentiate between two main approaches for generating landscapes. The first is a pattern-based approach, which uses mathematical algorithms to generate patterns regardless of the underlying processes [5]. Also referred to as “neutral landscape models” [1]–[3], [10], such an approach is explicitly and deliberately neutral to the biological and physical processes that shape spatial patterns. The second is a process-based approach, which aims to obtain certain spatial patterns as a result of hypothesized relevant processes [11]–[13].

### Pattern-based models: simplicity as a basis to advance theory

A simple map can be produced on the basis of a parameter *p* to determine the proportion of habitat cover, preferably in combination with a second parameter *H* to determine the degree of spatial autocorrelation or spatial cohesion. The most broadly used landscape generators in ecological studies are those based on algorithms derived from fractal geometry [3], [5], [13]–[19]. Among these, *Qrule* [10], [20] is particularly widely applied [2], [4], [19], [21], [22]. The power of these generators is their simplicity, combined with relatively high success in capturing a variety of realistic landscape patterns (e.g. [16]). Other pattern-oriented approaches exist as well. For instance, Wiegand et al. [23] developed a model which overlays a mix of Gaussian functions to create three-dimensional surfaces differing in ‘ruggedness’ (spatial autocorrelation), and then transecting them by horizontal planes to produce alternative thresholds of habitat quality. *Simmap* [24] is another neutral landscape model, which produces clustered patchy landscapes by assigning neighboring cells to the cell with the highest local density within a 3×3 neighborhood - an approach similar to Hiebeler [8], which applies a modal filter as in digital image processing techniques [24]. Another, newer generation of models attempts to reproduce also the spatial patterns that typify agricultural areas [25].

### Process-based generators: a tendency toward specificity

Process-based landscape generators are typically guided by a specific question, such as “what types of landscapes evolve given a certain process or parameters?” For example, Dale & Pearson [26] have shown that the ‘fishbone’ fragmentation pattern in the Amazon forest in Brazil emerges when a landscape is transected by roads, from which agricultural fields extend into the forest. Similarly, using the DISPATCH program, Baker [27] demonstrated that a set of decision rules for expansion of clear-cut logging areas into forests can successfully reproduce observed patterns of timber harvesting in subalpine forests in the USA. More sophisticated “land use cover change” models (LUCC) such as the “SLEUTH Urban Growth model” [28] and *Dinamica* EGO [29] offer the means to gain further realism and precision, and to reproduce a far broader range of spatial patterns. SLEUTH couples a cellular automaton with GIS data in order to predict urban expansion and associated expansion of agricultural lands for food production, using four “growth rules” alongside the capacity for learning (“self modification”). *Dinamica EGO* [29]–[31] is a grid-based model originally developed to mimic deforestation processes of small land-holders in the Amazon, but now used for a broad variety of purposes. It applies aggregated functions to mimic land-use changes by means of, e.g., transition matrices. Thus, *Dinamica* can be considered as a model which includes features of both pattern- and process-based landscape generator models. In a simple version it could also be used as a classical landscape generator, as in this study.

### Lack of simple models to mimic real patterns

When examining a range of landscape generators, we found pattern-based models to be simple and easy to implement, but often failing to reproduce spatial patterns that we perceive as typifying fragmented landscapes, such as stark boundaries between natural habitats and human-dominated areas. Process-based landscape generators seemed to produce highly realistic patterns but seemed too complex for generic application (especially when no input maps are available). The seeming lack of simple, process-based landscape generators for general purposes surprised us because one could speculate that a limited number of processes, namely the expansion of settlements, fields, and roads, likely dominate the spatial patterns of habitat loss and fragmentation in many regions of the world [32]–[38]. Consequently, we anticipate that process-based models could readily reproduce a wide range of spatial patterns, and yet serve equally well for explorative, generic purposes.

This study introduces a simple model that mimics the processes in which roads penetrate into natural environments, and landscapes are then transformed into agricultural fields (following Dale & Pearson [26]). Our model, *G-RaFFe*, therefore **G**enerates **R**oads **a**nd **F**ields for reproducing **F**ragmentation **e**ffects (an executable is available freely at www.ufz.de/index.php?en=21420). We delineate the model's concept, structure and parameters and then assess its performance by comparing its outputs, based on six indices describing landscape configuration, to 51 landscape maps from the Atlantic Forest in Brazil, Argentina and Paraguay. These landscapes range from 5 to 95% forest cover, and differ in fragmentation levels due to diverse land uses. We employ a similar approach to assess the performance of *Simmap* [8], [24] (random landscape generator), *Qrule* as a commonly-used fractal-based generator, and *Dinamica* [32], with which we developed four model versions differing in the number of processes and parameters included, from a pattern- to process-based approach.

## Methods

### The *G-RaFFe* model: concept and processes

Three main parameters govern the general behavior and end outcomes of our model: the desired habitat cover, the number of roads transecting the landscape, and field size, which reflects finer-scale determinants of the spatial structure. An additional parameter, ‘maximum field disconnection’, determines whether agricultural fields can be detached from roads or other fields, and if so, to which distance.

The model starts with a landscape of 100% forest cover. It then starts by generating roads, starting at any point along two of the four landscape edges and traversing the landscape in straight lines in one of three directions (straight, diagonally right or diagonally left), converting the forest into “non-forest”. Road lengths vary randomly along a uniform distribution (ranging from 1 to the diagonal landscape length). Roads are generated until the number of roads meets the desired number, unless, in the process, forest cover already reaches the target value determined by the user. Roads are one cell in width (unless defined otherwise, see below). Once all roads are generated, agricultural fields are extended from them. This process entails a random movement of simulated “farmers”, starting from any random point along the roads and moving one cell at a time, through the converted landscape (road or field), by choosing one of 8 neighboring cells at random. On meeting a forest edge, a field would be extended into the forest (unless the user defines that fields can be detached, in which case several further steps may be allowed, away from the road or field, before extending a new field). All fields have a quadratic shape (equal length and height), the size of which is taken from a uniform distribution between one and the maximum length determined by the user. Fields extend from the initial point, which is the field corner. Field expansion is a per-step process which may stop if the potential converted cells are beyond the map extent, or if the desired forest cover is reached. In the process, forest cells are converted into “open”, existing fields remain unchanged, but roads are not overridden until the last simulation step because they serve as seeds for field-creation and expansion. Finally, the eight cells surrounding the starting point are cleared of forest as well. This final process is required because, otherwise, forest cells remain uniformly scattered across the landscape. Forest cover is recalculated at each cell conversion so as to halt the simulation at the exact desired habitat cover.

### Input parameters and landscape generation

The main input parameters for the model are the 1) map extent; 2) the desired habitat cover; 3) the number of roads; 4) the maximum field size; and 5) maximum field disconnection, which would determine the number of steps in which random walk can be continued into the natural habitat before extending a new field. Potential additional parameters are: road width (the default value is 1); and the proportion of agricultural fields belonging to a second field type (e.g. plantations). In addition, for the systematic generation of landscapes along a continuum of habitat covers, one may determine a fixed relation between the desired habitat cover and the number of roads – to account for the fact that the number of roads alters with habitat cover. Based on a preliminary analysis, we identified an exponential relation between forest loss and the number of roads. The user can therefore determine the value of the parameters *a* and *b* in a function where the number of roads = *a•HC^b^*, where *HC* is the desired habitat cover.

For all models assessed in this study, we tried to cover the full range of spatial structures that can be generated by the model. We generated multiple landscapes, 256×256 cells in size (hypothetical cell size = 30×30 m, based on Landsat images), with habitat cover ranging systematically from 5% to 95% in increments of 5%. This extent and resolution was chosen to enable comparison with real landscape maps (see below).

For *Simmap* we varied the parameters *p* (the parameter that controls the degree of fragmentation of the obtained patterns) and *m* (minimum mapped unit = size of the smallest patch, determining the typical patch size), with nine values of *p* and seven values of *m* (see Table 1).

**Table 1:** Input parameters explored by the models *G-RaFFe*, *Qrule* and *Simmap*.

*Model*	*G-RaFFe*	*Simmap*	*Qrule*
Parameter	Number of roads	p	H
Values	(*a,b* = …)*[0.2,4]; [0.25,0.45]; [0.3,5]; [0.45,5]; [0.75,5]; [1.2,5]	0.05, 0.1, 0.15, 0.2, 0.3, 0.45, 0.52, 0.56, 0.59	0,0.05,0.1… 1 (21 values)
Parameter	Field Size	m**	
Values	5, 10, 15, 25, 40	1,2,4,8,16,32,64	
Parameter	Maximum field disconnection		
Values	0,1,3,5***		

Parameter values are provided for a given value of habitat cover.

* value of the formula y = *a·x^b^* to determine the relation between the number of roads and habitat cover.

** The parameter m is somewhat comparable to field size – see main text.

*** values used only for exploring the impact of the parameter. For comparisons between models and real landscapes, we used only the parameter values 0 and 3.

For *Qrule*, we altered *H*, the parameter which determines the level of spatial autocorrelation in the landscape (0 being close to random and 1 being completely clustered) systematically from 0 to 1 in increments of 0.05, resulting in 21 parameter values for each habitat cover.

For *Dinamica*, we developed four model versions of increasing complexity, where the simplest version (1) includes one process with 4 parameters to create spatially-independent patches (thus functioning like neutral pattern-based landscape generators), version 2 includes two processes (patch creation and a spatial-dependent patch expansion) with 8 parameters, and versions 3 and 4 include the same parameters and processes as versions 1 and 2, but additionally utilize an input road map which is produced as a first step using a built-in road constructor algorithm in *Dinamica* (additional 4 parameters). For further details on the explored parameters and processes for *Dinamica*, see Table 2 and Appendix S1).

**Table 2:** Processes and number of parameters included in the different model versions of *Dinamica*.

Process	# Param.	*Dinamica* Syntax	Model versions			
			1	2	3	4
Transition probability	1	Transition within transition Matrix	+	+	+	+
Patch creation	3	Functor: Patcher*	+	+	+	+
Patch expansion	4	Functor: Expander	−	+	−	+
Road creation**	4	Functor: road constructor	−	−	+	+
Additional param.***	9	Weights of evidence, friction and attraction map	w	w	w+	w+

The table delineates the processes included, number of parameters explored (# Param.), and how these were included in the different model versions of *Dinamica*. For convenience of replicating these model versions, we provide the syntax for landscape generation. For a full list of parameters and further guidelines see Appendix S1.

* *To activate patch creation processes go to the Dinamica function “Allocate Transitions”*.

** *Road creation was performed prior to patch creation and expansion*.

*** *weights of evidence (w) alone had a total of 7 parameters (see Appendix S1)*.

To characterize the impact of the different parameters of *G-RaFFe* on spatial patterns, we used six parameter combinations for the relations between the number of roads to habitat cover, five maximum field size values, and four maximum field disconnection values (Table 1).

For each parameter combination we generated 100 output maps, yielding a total of 182,000 maps for *G-RaFFe*, 119,700 for *Simmap*, and 39,900 for *Qrule*. Due to the larger number of parameters in *Dinamica*, we generated 50 output maps per parameter combination, resulting in 1,231,200 maps for version 1; 297,600 for version 2; 552,000 for version 3; and 1,623,800 for version 4 (Appendix S1). To enhance comparability between *Simmap*, *Qrule* and *G-RaFFe* in terms of the number of output maps compared to real maps, we used only 91,200 maps from *G-RaFFe* by selecting only two values of the parameter maximum field disconnection (0 and 3). Thereby, we reduced potential biases in performance which may emerge from differences in the number of landscapes or parameter combinations. Due to the large number of parameters in *Dinamica*, such a dilution procedure was not feasible.

### Landscape metrics

Many indices are available for describing landscape configuration and composition, yet the majority of these indices are highly correlated [9]. We chose six indices with low correlation, which together depict attributes of the overall landscape structure, the patches, and their spatial arrangement. These indices, calculated with Fragstats [39], were: 1) the total number of patches, 2) average patch size, 3) Largest Patch Index (LPI, expressed as a proportion of the landscape), 4) Average Euclidean distance between fragments, 5) Landscape Shape Index (LSI, calculated as the total length of edge cells divided by the minimum edge length possible if all cells were aggregated), and 6) Patch cohesion, an index which describes the level of clustering of patches in the landscape by calculating the perimeter-area ratio divided by the shape index of patches, both of which are computed as means weighted by patch area. This index was specifically designed for predicting habitat connectivity [40], [41]. We calculated the value of each of the six metrics first for all of the real landscapes and then for each of the landscapes generated by all models.

To characterize the behavior of *G-RaFFe* in terms of its acting parameters, we performed multiple and partial regression analysis to estimate how the number of roads, field size, maximum field disconnection and the target habitat cover (as independent variables) affect five of the landscape metrics (as dependent variables): the number of patches, LPI, LSI, average patch area and patch cohesion. To this end, we plotted the R^2^ of the multiple regressions and the partial R^2^ of the explanatory variables. We further evaluated the influence of model parameters on landscape variability, by performing simple regression analysis between each of the parameters. Here, we exclude field size and maximum field disconnection because these parameters have a secondary influence on the determination of landscape structure.

### Real landscape maps as a template for analysis

We selected 51 landscape maps from the Atlantic forest in Argentina, Paraguay and Brazil, ranging in forest cover from 5 to 95%, and covering a large range of spatial patterns due to variations in land use and land ownership between regions and countries (see Appendix S2). Landscapes were divided into three categories according to the dominant land use (Zurita and Bellocq 2010): 1) Small farms: these are landscapes typical to the Northeast of Misiones province in Argentina, where farm sizes usually range from 5 to 200 hectares. Main crops are tobacco and corn (annual), Yerba mate (*Ilex paraguarensis*, perennial) and small cattle pastures; 2) large farms: landscapes typical to southeastern Paraguay which are dominated by cattle pastures and soy-bean fields, occurring on large tracts owned by a small number of farmers; and 3) tree plantations: typical especially to northwestern Misiones, Argentina, where the main anthropogenic land uses include *Pine* plantations as well as *Eucalyptus* and *Araucaria* plantations, usually on large properties. The original maps, with a resolution of 30×30 m, were obtained by classifying Landsat TM images using an isodata, non-supervised algorithm with 20 classes, and then grouping the resulting classes into native forest versus five main land uses: annual crops, perennial crops, tree plantations, clear-cuts, and cattle pastures. This was based on spectral signature, IKONOS images, and field validation [42]. We cropped the original landscape maps to a size of 256×256 cells, and clustered all anthropogenic land uses into ‘forest’ and ‘non-forest’ for comparison with the maps generated by the different models.

### Comparisons between generated maps and real landscapes

As a first measure of the performance of the different models, we plotted the values of the six characterizing landscape indices produced by each model against habitat cover, visually comparing the range of model outputs with the range of values characterizing the real landscapes. This served as a single-criterion evaluation of performance. We then took two approaches for evaluation of performances against all metrics simultaneously. The first was based on a multivariate Principal Component Analysis (PCA), for each of 19 forest categories (5–95% in increments of 5%), including simultaneously both the real and simulated landscapes of each of the models (Appendix S3). We used the six indices as grouping variables, where each value represented the average of 100 generated maps for a given parameter combination. We then measured the Euclidian distance between each real and simulated landscape of the different models on the PCA bi-plot (using Axis I and II of the ordination), assuming that shorter distances indicate higher similarity of landscape structure between the simulated and real landscapes. We selected the best possible fit for each model (i.e., the single point among all parameter combinations which produced the shortest Euclidian distance to the point representing the real landscapes). For comparability between all models and an intuitive measure of performance, we inverted and transformed all Euclidian distances to a range of 0–1, 0 indicating low performance (high Euclidian distance) and 1 indicating highest performance (minimum Euclidian distance on the bi-plot).

The second analysis approach, which accounts also for the variability of landscapes produced for a given parameter combination, defines a matching of a given metric if the value of a real landscape lies within the range of values generated for that parameter combination (100 landscapes) – i.e., defining it as statistically indistinguishable from the generated set of landscapes [43], [44]. We then searched for the parameter combination that yielded the maximum number of simultaneously matching landscape metrics among all combinations, and registered how many metrics were matched and what model parameters yielded that best match.

For both analysis approaches, we performed a Factorial ANOVA using habitat cover categories (5–20%, 21–40%, 41–60% and >60%) and model type as independent variables, and model performance as the dependent variable. Thereby we assessed how performance changes between models, habitat cover categories, and the interaction between both.

Finally, we explored the relation between anthropogenic land use, landscape configuration, and the performance of each of the landscape generators. To this end, we divided the real landscape maps either according to forest cover or according to the most dominant non-forest land use (tree plantations, small farms, or large farms). To assess how dominant land use affects spatial patterns we performed ANCOVA with the six landscape metrics as dependent variables and dominant land uses as an independent variable. Forest cover was included as a co-variable since landscape metrics are highly dependent on it. To evaluate whether land use also affects the performance of the models, we performed a two-way ANOVA using landscape simulator and land use type as categorical variables, and model performance as the dependent variable.

## Results

### Characterization of *G-RaFFe*'s behavior

**Figure 1:**
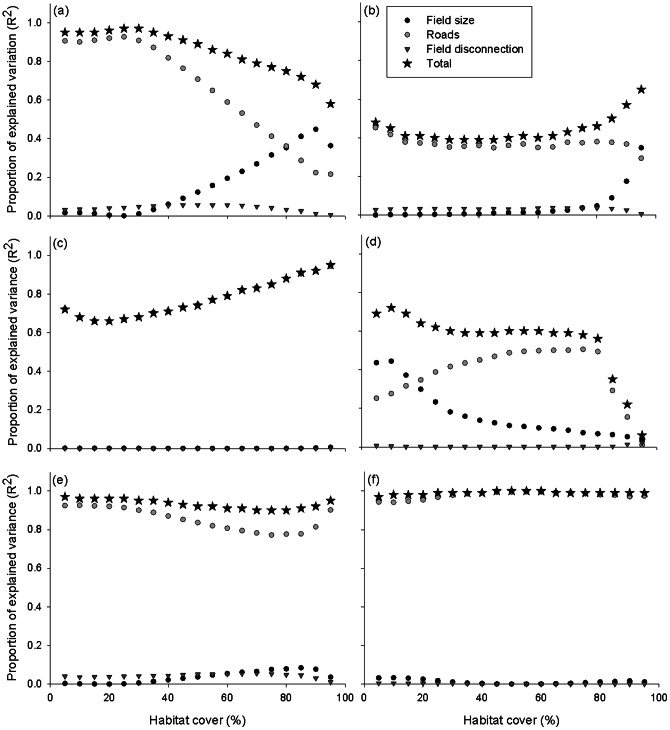
Strength of effect of *G-RaFFe*'s main parameters on landscape attributes. Multiple regression analysis and partial regression analysis between model parameters (number of roads, field size and maximum field disconnection) and a) the Number of patches, b) Average patch size, c) Largest Patch Index (LPI), d) Euclidian distance between patches, e) Landscape Shape Index (LSI), and f) patch cohesion.

Figure 1 and Table 3 depict the behavior of *G-RaFFe* in terms of the strength of effect of each of its acting parameters (number of roads, field size, and maximum disconnection) on spatial patterns according to the six landscapes metrics. We found an overall strong effect on the number of patches, LSI, LPI and cohesion (R^2^>0.6, p<0.001 in all cases) and a medium influence on the Euclidian distance between patches and the minimum patch area (0.4<R^2^<0.6, p<0.001 in all cases). Partial regression analysis indicated the number of roads as the main determinant of landscape attributes whereas the influence of maximum field disconnection was very small (Table 3). Maximum field size influenced the number of patches and minimum patch area at high values of habitat cover (Figure 1a and f), and the average distance between fragments at low habitat cover (Figure 1e).

**Table 3:** Results of Multiple Regression to assess sources of model variability in *G-RaFFe*.

	*Parameter coefficients*			*Forest cover*		*Number of roads*	
	*Roads*	*Field size*	*F.Dis.*	Direction of effect	R^2^	Direction of effect	R^2^
Number of patches	0.51	0.16	−0.17	Increase	0.92*	Increase	0.85*
LPI	−0.57	−0.001	0.004	Decrease	0.98*	Decrease	0.13*
LSI	0.61	0.17	−0.11	Positive	0.99*	Positive	0.83*
Area	−0.3	−0.09	0.16	Positive	0.99*	Positive	0.83*
Euclidian distance	−0.1	0.025	0.28	Positive	0.51*	Positive	0.01
Cohesion	−0.97	−0.03	−0.06	Decrease	0.97*	Decrease	0.66*

We depict the parameter coefficients of the acting parameters Road, Field Size and Maximum Field Disconnection (F.Dis.) as well as the direction and strength of effect of the two most important factors, namely forest cover and the number of roads.

* = P<0.05.

### Comparative performance of the models

**Figure 2:**
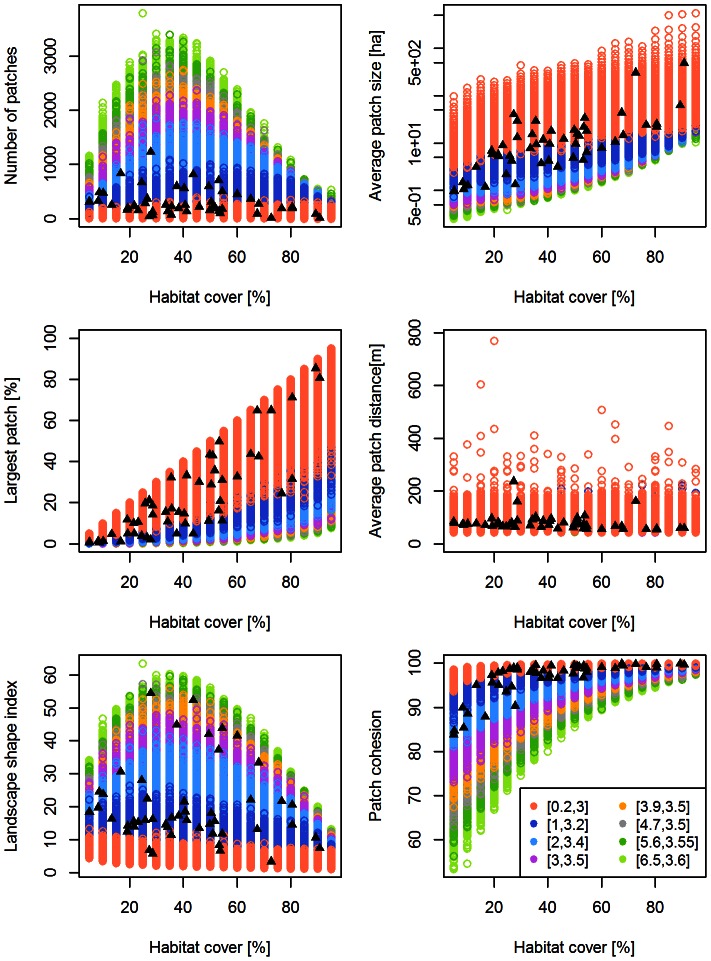
Attributes of the landscapes generated by *G-RaFFe*. Generated landscape parameters are illustrated in colored circles, and compared to 50 real landscape maps (full triangles) according to six explored landscape attributes, each against habitat cover: a) Number of patches, b) Average patch size, c) Largest patch index, d) Average distance between patches, e) Landscape Shape Index, and (f) Patch cohesion. The colors represent the effect of the number of roads, expressed by the combination of parameters (*a,b*) that determines the relation between habitat cover and the number of roads. Overlaps between parameter outputs cannot be seen due to color dominance.

**Figure 3:**
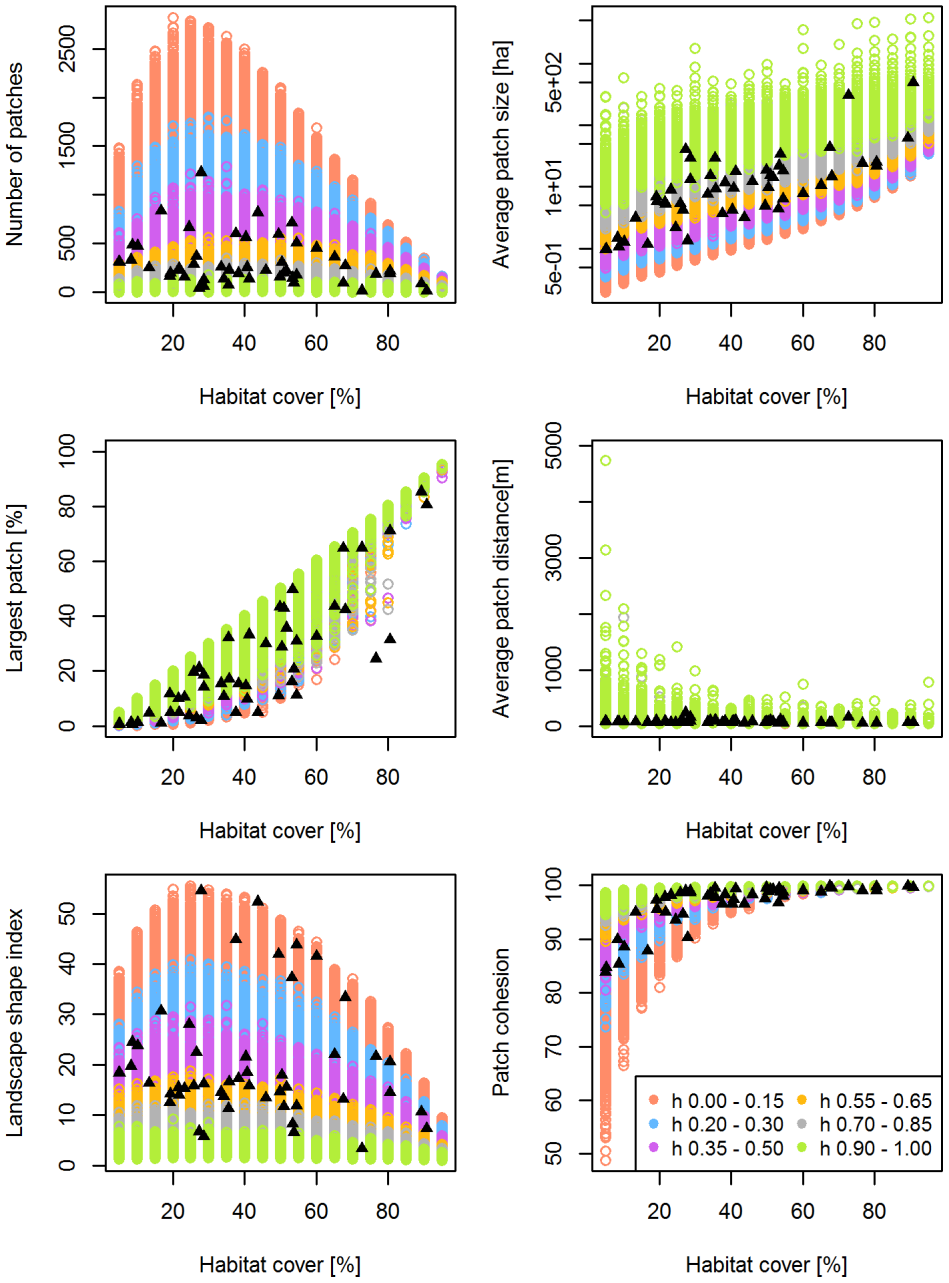
Attributes of the landscapes generated by *Qrule*. Generated landscapes are illustrated in colored circles, and compared to 50 real landscape maps as in Figure 2. Colors represent the varied value of the Hurst exponent (*H*), defining the clustering coefficient.

**Figure 4:**
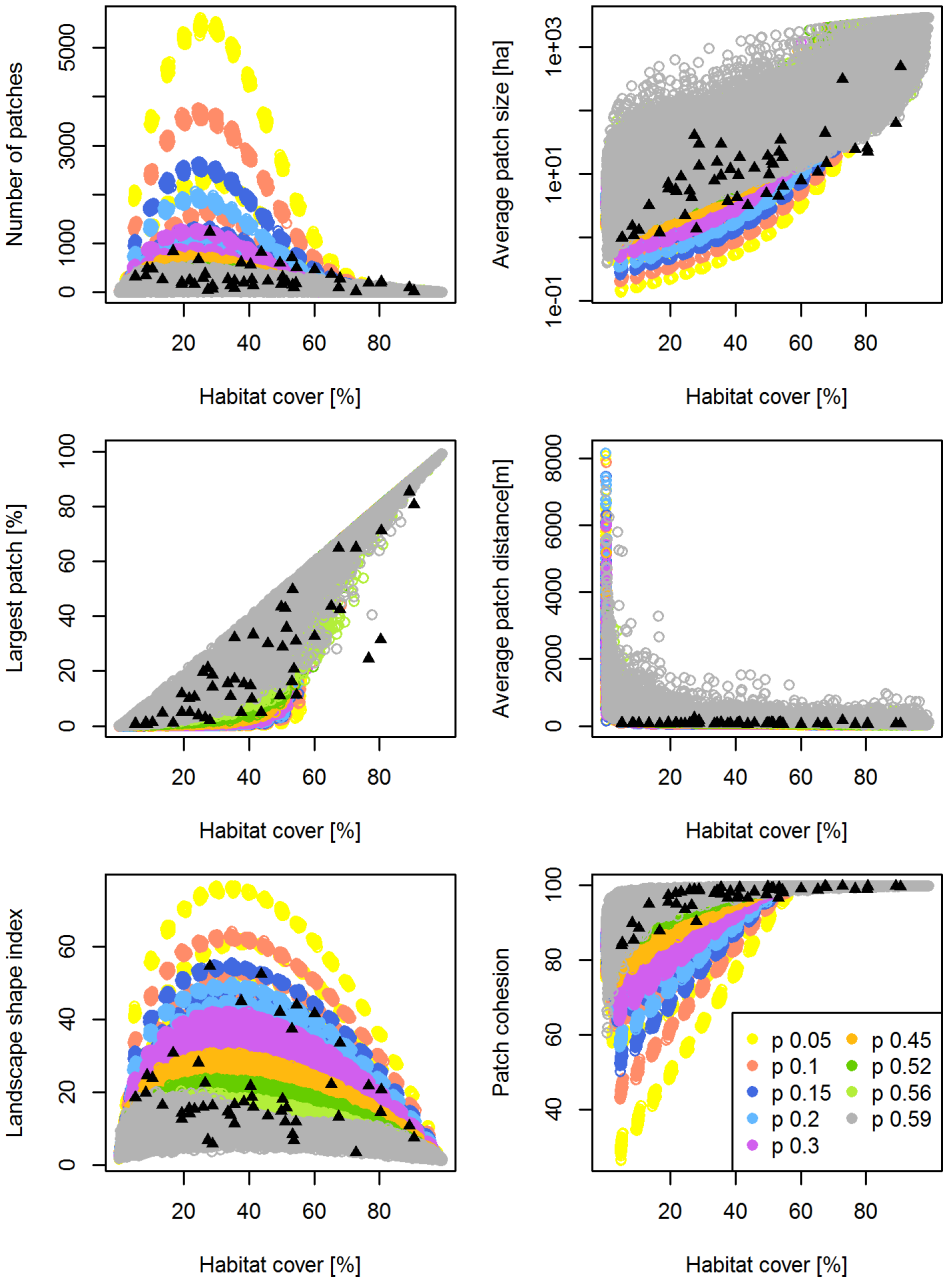
Range of attributes of the landscapes generated by *Simmap*. Generated landscapes are illustrated in colored circles, and compared to 50 real landscape maps as in Figure 2. Colors represent values of the parameter *P*, the parameter that controls the degree of fragmentation of the obtained patterns.

**Figure 5:**
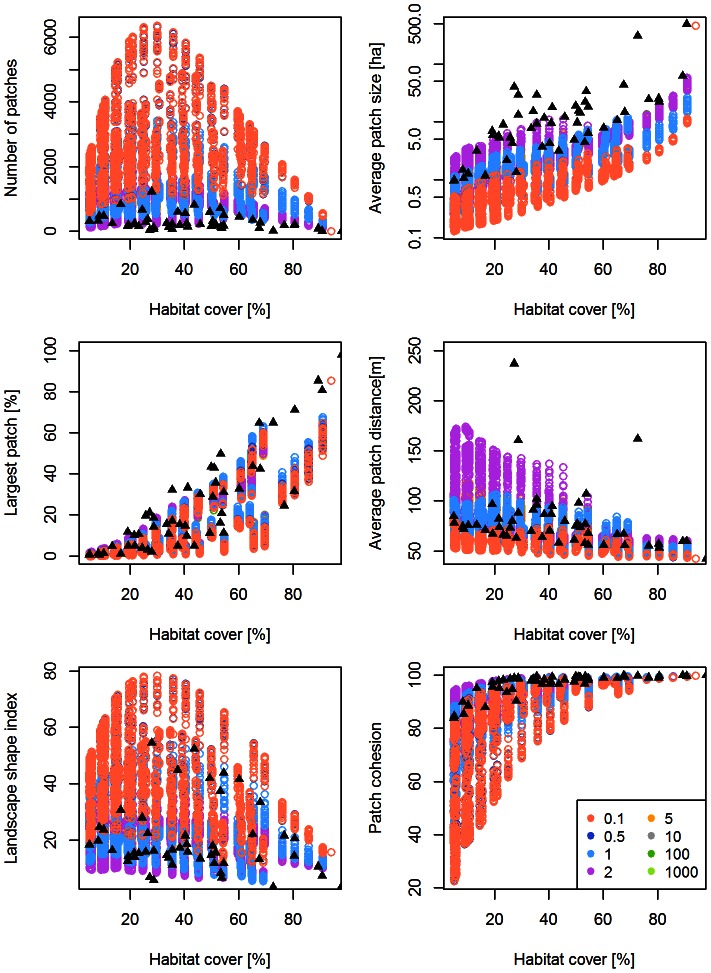
Attributes of the landscapes generated by *Dinamica* (version 4). Generated landscapes are illustrated in colored circles, and compared to 50 real landscape maps as in Figure 2. Results illustrate *Dinamica* in the most complex model version 4. Colors represent values of the parameter *patch generation parameter 3*, the parameter that controls patch isometry. For model versions 1–3 see Appendix S1.

For a single-metric comparison between the models and the real landscapes, we found that *G-RaFFe* produced landscapes that engulfed the full range parameters of all real landscapes, with values far beyond the range of the inspected real landscapes in terms of the number of patches and patch cohesion, but only slightly higher than the range of average distance between patches (Figure 2). *Qrule* covered most of the range of real metric values, but some real landscapes were beyond the range of generated ones in terms of LPI and patch cohesion, or just at the edge of the range in terms of LSI (Figure 3). *Simmap* covered the full range of parameters for four metrics, but some real landscapes deviated from the range of generated lands in terms of average patch size and LPI (Figure 4). Additionally, values of the average patch distance greatly exceeded the range of realistic distances. *Dinamica*, at all model versions, produced a broad range of landscapes but mostly with an exceeding number of patches, primarily due to multiple single-cell patches remaining in the landscape. Consequently, many real landscapes remained unmatched in terms of the number of patches, average patch size and the largest patch index (Figure 5 for version 4; see Appendix S1 for versions 1–3).

**Figure 6:**
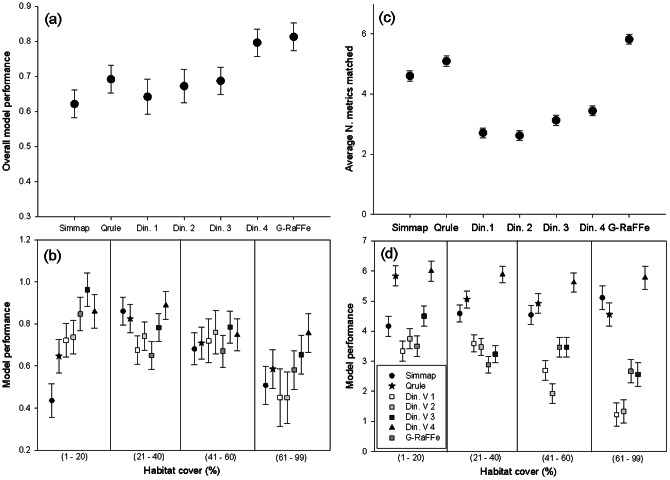
Performance of the different models in terms of their capacity to match real landscape maps. A,b: Results based on a PCA-based approach; c,d: based on the number of parameters that were simultaneously matched. Results are provided for overall model performance (a,c) and separated according to habitat cover categories (b,d). Models are organized based on complexity and based on the processes included, from left to right. Values reflect average values (± SE). Din. V = *Dinamica* version.

Multi-criteria evaluation of the models based on Principal Component Analysis revealed that overall model performance varied among simulators in terms of the capacity to reproduce realistic spatial patterns (Factorial Two-way ANOVA, F_6,308_ = 3.27, p<0.001), with *G-RaFFe* and *Dinamica* version 4 showing high and nearly similar performance, followed by *Qrule*, *Dinamica* versions 3, 2 and 1, and finally *Simmap* with lowest performance (Figure 6a). *Dinamica*'s performance hence increased with complexity, yet with a stepwise increment between versions 3 and 4, namely, when roads served as a seed to patch expansion. We found an interaction between simulator performance and habitat cover, each model changing differently in performance among habitat cover categories (Factorial Two-way ANOVA, F_18,308_ = 1.67, p = 0.04) (Figure 6b). Specifically, all models performed somewhat equally at habitat covers of 20–60%; *G-RaFFe* and *Dinamica* 4 performed better at habitat cover >60%; and *Dinamica* version 4, followed by *G-RaFFe*, had the best performance at habitat cover <20%.

Analysis based on the number of parameters fitting simultaneously verified that performance differed between models (Factorial Two-way ANOVA, Model: F_6,308_ = 134.1, p<0.001) but *G-RaFFe* was found to perform better than all other models, followed by *Qrule*, *Simmap*, and finally *Dinamica* versions 4, 3, 1 and 2 (Figure 6c). Furthermore, a successful matching of all 6 metrics was obtained by *G-RaFFe* for 41 of 51 landscapes (80.4%, with all other maps matched by 5 metrics. *Qrule* fitted 48% (with scores ranging from 2 to 6), *Simmap* 14% (ranging from 0 to 6), *Dinamica* version 4 matched two (3.9%) and versions 1 and 2 fitted only one (2%). We found again a significant interaction between model and habitat cover (Factorial Two-way ANOVA, Interaction: F_18,308_ = 3.34, p<0.001): Habitat cover had a minor effect on the performance of *G-RaFFe*, but a significant effect on all other models (Figure 6d). Particularly, *Qrule* had its best performance at habitat cover <20%, and *Simmap* at habitat cover >60%. For *Dinamica*, the addition of an input road map enhanced the performance of versions 3 and 4 at medium or high habitat cover (>41%). At habitat cover <20%, version 4 performed better than all other *Dinamica* versions.

### Relation between spatial patterns and real land uses

We found a significant relation between the dominant anthropogenic land use and the spatial patterns of the investigated real landscapes (Table 4). Landscapes comprising primarily of small farms had a larger number of remaining forest patches (independently of forest cover, ANCOVA), substantially lower LPI, and higher LSI due to the irregular structure of farms and forest patches. Landscapes dominated by tree plantations had a small number of remaining forest patches, lower LSI but high LPI, resulting from a regular spatial structure and the presence of corridors (thus reducing the number of patches and increasing edge length). Finally, landscapes dominated by large farms differed from those with tree plantations in terms of LPI, and differed from those with small farms in terms of the number of patches and LSI – namely, forming irregular spatial patterns but with a small number of patches (Table 4).

**Table 4:** Relation between dominant anthropogenic land use and spatial pattern.

	*Tree plantations*	*Large open areas*	*Small farms*	*F (p)*
Number of Patches	178^a^	284^a^	522^b^	5.8**
LPI	31.5^a^	22.3^b^	15.2^b^	3.9*
LSI	11.3^a^	19.3^a^	35.2^b^	12.5**
Area	232.9	71.1	153.5	2.3
Euclidian distance	77.9	84.1	72.8	0.51
Cohesion	96.1	97.6	95.8	1.7

Values represent the average value of each landscape metric for a given landscape type, significance marks the outcomes of ANCOVA with habitat cover as a covariate (* = p<0.05, ** = P<0.01). Allocation into groups (a,b) is based on Fisher's post-hoc analysis.

**Figure 7:**
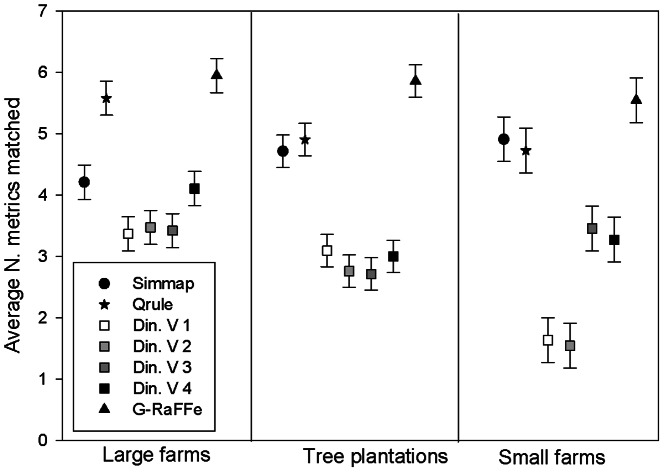
Effect of dominant land-use type on landscape simulator performance. Values represent the average number of landscape metrics that were simultaneously matched (± SE). Din. V = *Dinamica* version.

PCA-based analysis did not indicate an effect of these spatial difference on model performance (Two-Way ANOVA; F_2,315_ = 2.1, p = 0.11). However, assessment based on simultaneous matching of metrics found clear relations between model performance and landscape characteristics (Two-Way ANOVA; F_2,315_ = 9.4, p<0.001): *Dinamica* version 1 showed decreased performance at landscapes dominated by small farms compared to others, *Qrule* and *Dinamica* version 4 showed an increased performance in landscapes dominated by large farms compared to other landscape types, *Dinamica* version 3 showed decreased performance in landscapes dominated by tree plantations. *G-RaFFe*, however, did not show a significant change in performance across land (Figure 7).

**Figure 8:**
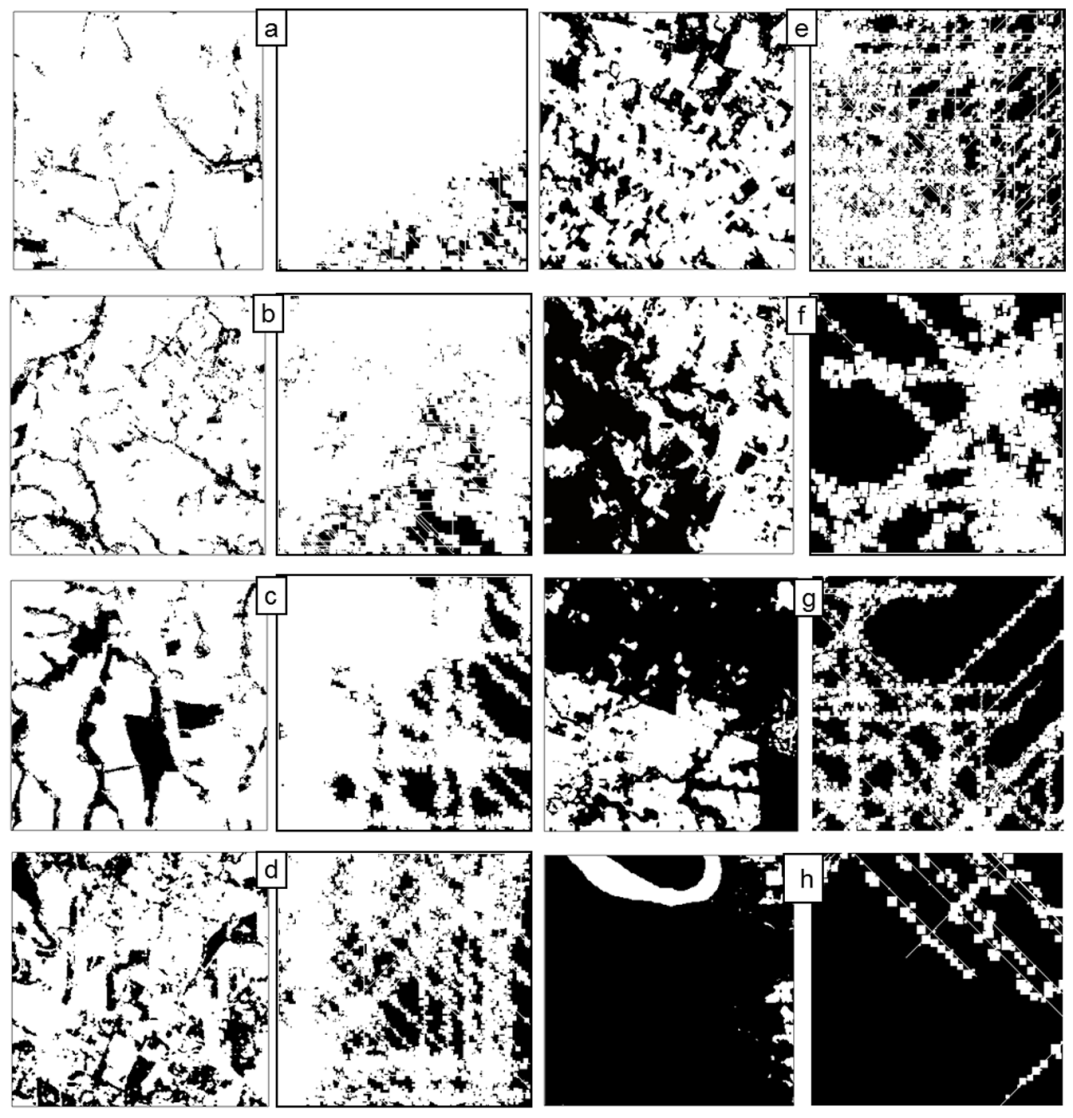
Examples of eight real landscape maps compared to corresponding landscapes generated by *G-RaFFe*. Depicted maps have a forest cover of 5, 10, 19, 26, 27, 51, 65, and 90%. Each landscape is compared to a corresponding landscape map generated by *G-RaFFe* with the parameters that were identified to provide the best match. Figure 8e (27% FC) was matched by *G-RaFFe* for only 5 metrics, and 3 for the other models; d,e,g,h were poorly matched by *Qrule* (<5 metrics matched), while *Simmap* failed for maps a,b,e,f (matching = 0) and g. For maps d,g, and h *Simmap* performed better than *Qrule*. For the parameters of the real and virtual landscapes see Appendix S2.

Finally, to illustrate the outcomes of *G-RaFFe*, Figure 8 depicts a selection of eight real landscapes compared to eight landscapes generated by the model, with the parameters identified as producing the best match. A visual inspection of the maps indicates a capability of generating spatial patterns that somewhat resemble real ones, but clear visual deviations do emerge: Large fields produce overly straight structures (Figure 8f), the square form of fields cannot form a parallel structure to diagonal roads (which is mostly evident at high forest cover, Figure 8h), and natural long elements such as rivers cannot be reproduced (Figure 8h). For one of the selected landscapes, *G-RaFFe* performed less successfully with only 5 matches (Figure 8e), yet for the same landscape, all other models matched fewer metrics. Comparison to *Qrule* and *Simmap* indicates that *Qrule* performed poorly for landscapes that are characterized by sharp boundaries between forest and non-forest (Figures 8d,e,g,h), and *Simmap* performed poorly when landscapes had, for instance, high variability in patch size (Figure 8a,b,e,f,g – for values see Appendix S3). For illustrations of *Dinamica*'s output maps, see Appendix S1.

## Discussion

This paper demonstrates the potential power of process-based landscape simulators for various virtual landscape patterns, with the benefit of reproducing spatial patterns that typify habitat loss and fragmentation in rural, agricultural, and forestry-dominated landscapes. When applying a set of parameters that are intuitive and resemble realistic processes, users can easily control the spatial attributes of the generated landscapes. Despite the simplicity of the generator we introduced, its outputs covered a wider range of spatial structures and performed better in matching realistic spatial patterns compared to pattern-based models of somewhat similar level of complexity. A *Dinamica* model version with somewhat same processes performed equally well according to a PCA analysis, and produced maps that seemed visually realistic (Appendix S1), but performed far more poorly with respect to matching criteria despite a higher number of parameters (Table 2) and a much larger number of landscapes produced and analyzed. This was primarily because of the low variability produced by *Dinamica* for a given parameter combination – something which may be desirable when seeking high predictive power, but not necessarily when seeking to maximize the range of generated spatial patterns. Exploration and implementation of *Dinamica* also required high effort in terms of resources, both in working time and technical infrastructure. This was due to the non-trivial GUI, the lack of immediate outputs, and a large number of parameters to explore. Furthermore, model versions where roads and fields extended in an iterative process (to mimic the expansion of a deforestation frontier) performed very poorly (see Appendix S1). We do not doubt that further calibration could yield better performance, but this is beyond the scope and purpose of this paper as here we attempted to inspect the performance of uninformed landscape generators.

Model complexity and the use of input maps can clearly yield high predictive power for specific case studies (examples for *Dinamica*: [30], [45], [46]; SLEUTH: [12], [47]–[50]), but at the cost of data-hunger, long preparatory stage for parameterization and calibration, and long calculation time. These findings suggest that the efforts to support a broad range of options and capacities, offered by a very strong software packages such as *Dinamica*, do not support the simplicity which is wished for when generating landscapes for explorative or theoretical purposes. Thus, a powerful aspect of process-based generators remains overlooked, namely their ability to mimic complex spatial patterns using realistic processes that can be simple, intuitive, and easy to communicate (see also [51]). An interesting result in this context is the leap in performance of *Dinamica* from model version 3 to 4, namely when incorporating both roads and road-related patch-expansion. This result confirms the central role of roads, especially as a starting point for agricultural expansion, in forming spatial fragmentation patterns (see also [38], [45], [52]). Another important lesson emerges from the fact that, unlike many other models, *G-RaFFe* produces fields that are clean of natural habitat. That this yielded high performance demonstrates the unique attribute of human-dominated land-uses, namely, the rarity or absence of natural features apart from those remaining along property margins (see also [25] for purely agricultural lands). Similarly, most pattern-based models for generating fragmented landscapes tend to overlook the fact that human activities often result in a non-random association of land-cover types [10], with sharp contrast between patches of natural areas and neighboring, often homogeneous anthropogenic land types [53]. Human-dominated areas are also usually more clustered than the remaining natural habitats, and structurally better connected through infrastructures such as roads. In consequence, the spatial patterns of anthropogenic landscapes is inherently non-fractal [16]. We therefore suggest great caution in the selection of neutral landscape models, even if used for theoretical purposes, to ensure that the templates used for analysis suit the spatial structures of the system in question. In this, we reiterate Halley et al. [54] in calling for careful application of fractal approaches to patterns that may be non-fractal.

### The range of applicability of the approach

The relative performance of the different models clearly altered with habitat cover and the dominating land-use type, yet *G-RaFFe* seemed to perform equally well across fragmentation levels and land-use types, and often better than the other tested models. This predictive capacity was not anticipated *a priori* when developing the model, or even when visually inspecting the output maps. Yet we validated that our results are robust to the selection of metrics or analysis approach. We therefore attribute them to the fact that the models differ in capacities, weaknesses, and realms of potential applicability. Likely, *G-RaFFe* is most suitable for reproducing the spatial patterns typifying regions undergoing habitat loss and fragmentation, or ‘marginal landscapes’ where human accessibility is limited – namely, those landscapes that warrant particular attention due to human pressures on biodiversity. On the other hand, the model may be less suitable for investigating gradients within natural or semi-natural environments, or for regions already dominated by anthropogenic infrastructures, such as urban areas dominated by agricultural fields with little or no remaining natural areas. In such cases, we suggest using other landscape generators (e.g. [5], [10], [22], [23], [25], [29]).

The scale and resolution of interest may affect model performance and applicability as well, since some landscape elements occur at some scales but not at others. For example, forest gaps due to selective logging or natural processes [55], [56] can be visible only at fine resolution, and linear elements tend to disappear when increasing map extent and reducing its resolution – starting with hedge rows and small roads, followed by streams, and even rivers are lost at coarser scales [57]. Accordingly, the power and applicability of different landscape generators, the role of their different parameters and their potential capacity to identify spatial signatures of drivers, are likely scale-dependent [58].

### Model versus reality: process-based models as potential tools for learning

Complex socio-economic processes shape deforestation patterns (or more generally, landscape structures), and these are poorly known [10]. Thus far, advanced methods for analysis and simulation have failed to rigorously link spatial patterns with processes ([10], but see [59]). Mathematical approaches can potentially generate such spatial patterns but are unlikely to further our understanding of the processes that generate them. Process-based approaches can therefore facilitate analyses of the relation between processes and spatial patterns. For example, in this study we found landscape accessibility (here, number of roads) to determine the number of remaining forest patches and their size (for empirical evidence of such a relation see, see e.g. [34], [37]), and was a major determinant of the Landscape Shape Index (LSI) which represents the length of edges in a landscape. But “field size” determined had an important effect of the spatial configuration of patches, such as the average distance between patches. It further determined the number of patches and their structure in landscapes with high habitat cover (i.e., early fragmentation stages).

We see particular value for studies aiming to a) maps and quantify the number and spatial distribution of roads across landscapes [38], as well as b) to gain better understanding of the parameter “field size”, which relates to patterns of land ownership and management (and hence to socioeconomic factors). This is shown by the matching between the dominant land use and the corresponding field size that was found to best match them. Further empirical evidence of such a relation was recently provided by two case studies from Brazil [52], [53].

### Prospects for further model development

Some potential improvements of *G-RaFFe* warrant discussion here. The addition of some parameters may enable model use for further purposes, albeit with a potential loss of generality, or at least of simplicity. Examples include:

1. Break the regularity of fields and the straightness of roads, to yield a visually more realistic pattern (see Appendix S1 for *Dinamica* results, and [27]). Fields can expand from a central point outwards, whereas roads can be formed through a correlated-random-walk and emerge from one another. Some of these aspects are already included in a new model version of *G-RaFFe*, and some are under construction. Nonetheless, we will attempt to do so with a minimum number of parameters.
2. The spatial patterns produced in this study are mostly typical to flat terrains. To allow the generation of landscapes that resemble those of topographically more complex landscapes, one can use real or mathematically-generated landscape terrains (see e.g. [23]), in combination with a preference for generating roads and fields on flat grounds or along elevation isoclines (i.e., defining a “slope resistance” [28]).
3. Finally, one may wish to add corridors to the landscape produced, e.g. to test their potential effects on functional connectivity. *G-RaFFe* already offers this option using a sub-module that forms several randomly placed stretches of natural habitat (forests) on top of the road-transected map, prior to the expansion of fields. Advantages of this procedure are that a) it does not require *a-priori* knowledge of where patches are, but instead mimics the presence of a physical barrier; b) it yields relatively irregular corridors; and c) it enhances connectivity without major alteration of the (visual) spatial structure from an alternative map without corridors.

The *G-RaFFe* model has been used in some ecological explorations [6], and current applications are on-going. The model can be downloaded freely at www.ufz.de/index.php?en=21420. As with any other model, its use may give rise to criticism and suggestions for improvement. We welcome all comments or suggestions, as they will certainly contribute to the development and application of the model.

## Supporting Information

Appendix S1 **Description and implementation of Dinamica EGO landscape generator.**
(DOC)

Appendix S2 **Comparison of the attributes of the real and simulated landscapes for the landscape generators G-RaFFe, Qrule and Simmap.**
(DOCX)

Appendix S3 **Principal Component Analysis for the performance of all models in reproducing realistic spatial patterns.**
(DOCX)
