# Supplementary material for: Simple Process-Based Simulators for Generating Spatial Patterns of Habitat Loss and Fragmentation: A Review and Introduction to the G-RaFFe Model
Source: PLoS One. 2013 May 28;8(5):e64968. doi: 10.1371/journal.pone.0064968 (PMC3665680; doi:10.1371/journal.pone.0064968)
Supplement: Appendix S1 — Description and implementation of Dinamica EGO landscape generator. (DOC) [file pone.0064968.s001.doc]

**Appendix 1:**

**Description and implementation of Dinamica EGO landscape generator**

Contents

[1 Model description 1](#__RefHeading___Toc353522164)

[2 Simulation Experiments 5](#__RefHeading___Toc353522165)

[3 Results (not occurring in the main text) 7](#__RefHeading___Toc353522166)

[4 Hints for implementation of Dinamica for landscape generation 8](#__RefHeading___Toc353522167)

[5 References 9](#__RefHeading___Toc353522168)

[6 Tables 11](#__RefHeading___Toc353522169)

[7 Figures 15](#__RefHeading___Toc353522170)

## 1 Model description

The following sections roughly follow the ODD protocol (Overview, Design concepts, Details) (Grimm et al. 2006, 2010), which was originally designed for describing individual- and agent-based models. The protocol consists of seven elements: The first three provide an overview, the fourth provides general concepts underlying the model’s design, and the remaining three elements provide details, which we allocate for further explanation on submodels and model implementation.

**Purpose -** The purpose of using the modelling environment Dinamica EGO (Soares et al. 2002, 2004, 2009) was to test the capacity of the program to develop spatial maps of deforestation patterns, either through pattern-based or process-based algorithms for landscape generation.

**Entities, state variables, and scales -** We developed four model versions: two pattern-based ones (Model 1 and Model 2) which contain only the state variables “forest” versus “deforested”, and two process-based models (3 and 4) that include also the state variable ”road”. All maps produced had an extension of 256 × 256 cells, and a cell size of 30 × 30 m.

**Process overview and scheduling -** Dinamica includes three processes that are comparable to *G-RaFFe*: patch creation, patch expansion, and road expansion. In the simplest model version (hereafter, Model version 1), the only acting process is *patch creation*. Patches are created without a spatial structuring rule, as in a fully neutral modelling approach. Model version 2 includes two processes: one for *patch creation* and one for *patch expansion*, the latter being spatially aggregated. With both acting processes, the model still can be regarded as pattern-based, as it functions somewhat similarly to *Qrule* and *Simmap*. To obtain a stronger relation to realistic spatial patterns, Models 3 and 4 use an input map containing a road network which is produced prior to patch expansion, using a “*road constructor*” model (Soares et al. 2004). Model version 3 applies the non-spatial process of *patch creation* only (analogically to Model version 1), whereas model version 4 includes both processes, *patch creation* and *patch expansion* in differing proportions, analogically to Model version 2. Dinamica also enables running the road expansion and patch expansion processes in an iterative way, thus mimicking a gradual expansion of a deforestation frontier. This option was implemented in preliminary simulations (see below, model versions 5 and 6), but not included in the main text of the paper. For further details on the processes see section “submodels” below.

**Design concepts**

For *Basic principles* see above.

The main *emergent* result is the remaining forest cover and its spatial distribution, as well as the correspondent land-use map, after *n* iterations (time steps). This value depends on the amount of area which is deforested per iteration of patch creation or patch expansion, as determined primarily by the transition matrix which defines the amount of transition from one class to another. The decision where to create a patch depends mostly on a ‘spatial transition probability map’, which defines the spatial allocation of deforestation probabilities based on distance between land-use types (e.g. distance of forest cell from a road or deforested area), referred to as “distance depended allocation of patches”. To derive this map, Dinamica calculates the frequency of distances between land-use types, applying a “weights of evidence” method (Soares et al. 2009).

*Stochasticity* in the model is included in all processes by applying probability and rule-based approaches. For further details see “submodels” below and Soares et al. (2004, 2009).

Potential *observations* produced by Dinamica include the remaining relative forest cover (an outcome of the number of iterations). Output maps can be saved along the simulation process to obtain a series of outcomes over one simulation. However, maps are not produced and depicted automatically, meaning that visualization of the results can only be achieved once a simulation is completed. Spatial analysis of landscape metrics requires other tools such as Fragstats (see main text). To view the many ascii maps produced by Dinamica, we wrote an ascii-viewer that enables effective assessment of series of maps.

**Initialization -** The model starts with uploading input maps and the “weights of evidence” file which are necessary for any model applying distance-dependent probabilities. It then calculates the transition probability map prior the simulation.

**Input data –** Required input data include an initial land-use map, the set of input parameter values as determined by the user (Table S2), and an additional file containing the “weight of evidence” parameters (Table S3). To run the *road constructor*, additional two maps are necessary: a friction map and attraction map (see “submodel”). We did not use real landscape maps as inputs, but instead used an input map with 100% forest cover for model versions 1 and 2. For those versions using roads as an input for patch creation and expansion (Model versions 3 and 4), a road seed is usually required. To produce it, we created an input map applying a single transition of all cells along the northern and western rims into a road. One cell was then randomly selected as the initial seed, producing a map with 100% forest cover minus one road-cell as a seed. Dinamica also requires a file defining map size, cell size, and cell state categories (in our case only “forest” or “non-forest”, and for versions 3-6 also “road”).

**Submodels -** In the following we briefly explain the standard processes of Dinamica and especially how they were used in this in this study. For detailed description of the algorithms see the introductory handbook (Soares et al. 2009).

*Patch creation -* This algorithm creates patches of aggregated small elements with defined size until the possible transition area is filled up with patches. It is controlled by the parameters “mean patch size”, “patch variance”, and “patch isometry”. Randomness in patch size is allowed by the parameter patch variance, while the isometry parameter steers the form of the patches, from less isometric (e.g. linear) (0) to more isometric (>1, namely, more quadratic or circular) elements. For more details see Soares et al. (2009).

*Patch expansion -* The expansion process enlarges already existing patches to the size defined by the size parameter. The set of governing parameters are the same as in *patch creation*. The relative share of patches created by *patch creation* or *patch expansion* is determined by a moderating parameter “*Per-cent patch creation by expansion*” (Table S1). For more details see Soares et al. (2009).

*Road creation –* This tool creates roads in a stepwise process, one linear object at a time, starting at one initial deforested point and later on starting at any point along a previous road. Road directionality and the form of the road network are controlled by four parameters (Table S1): i and ii) Shorter and longer length along which a new branch is created (meaning the minimum and maximum possible distance between two branches), and iii) and iv) number of horizontal and vertical quadrants (meaning, to our understanding, that the landscape is divided into virtual cells of equal size, defining the potential maximum expansion of one road within one iteration). Further, one can influence road creation by providing non-neutral values for the additional input friction maps defining areas of high costs to build a road, or attraction maps defining points of high attractiveness toward which roads tend to be created. In this study, both input maps were set to uniform values throughout. For more details see Soares et al. (2004).

## 2 Simulation Experiments

We developed six model versions (**Table S1**), four of which included in the main text of this study as mentioned above: Model version 1 contained only the non-spatial process “*patch creation*”; Model version 2 included additionally the spatial-dependent process “*patch expansion*”; Model version 3 contained an input road maps and used only the “*patch creation*” process, and model version 4 included additionally the spatial dependent process “*patch expansion*”. Illustrative examples of outputs of these four models are provided in **Figure S1**.

In addition, a potentially better simulation process would be one which allows roads to expand in parallel to field expansion, or at least as an iterative process as demonstrated by the illustrative maps below (**Figure S2**). However, such a procedure entails several additional parameters and complexities, and yielded poorer results (see below). Hence, these maps were not included in the main body of this paper.

Each model version was performed with an extensive parameter variation in order to generate as broad a range of spatial structures as possible, similarly to the exploration of *G-RaFFe*, *Qrule* and *Simmap* (see **Table S2** for parameters and their explored ranges/values). We did not vary the parameters of “weights of evidence”, as this would have required a substantial number of additional working-days for parameterization and calibration. Instead we utilized the weights of evidence values provided by the “patterns of change” example within Dinamica (Soares et al. 2009, example 9; for values see **Table S3**).

Forest cover cannot be predetermined directly by the user, as it depends on the number of time steps and the speed of deforestation processes applied. To generate a range of forest covers we therefore initially varied the number of time steps for testing after how many iterations the lowest forest cover (5%) is reached. The number of time steps was then predefined based on these preliminary simulations. During the main simulations, we saved output maps along the process, to generate a series of maps for each simulation and each parameter combination with forest cover of approximately 95, 90 etc. down to 5%. We additionally developed a new tool, namely a filtering algorithm (externally to Dinamica) to inspect forest cover during runtime and accept landscapes for saving if falling within proximity of ± 1% to the desired habitat-cover category. Thereby, we rejected approximately 60% of the maps produced by Dinamica (see **Table S4**). Each parameter combination and the production of a series of maps were repeated 50 times.

**Simulation running and first analysis**

To explore multiple parameter combinations without exploding the number of generated maps, we fixed some parameters which in preliminary simulations (for Model version 1) have shown only weak impact on simulation outcomes. Nonetheless, the overall number of simulations, with 100 repetitions per parameter combination and 4 model versions, necessitated generating millions of maps. Dinamica enables multiple simulations to run using the command line, but only for a given parameter value and a single computing node (core). To overcome this barrier, we wrote a C++ code which reads parameter variations from a database and writes it into the Dinamica input file. To this end, Dinamica was installed on multiple nodes.

Maps that fitted the essential forest cover criteria were kept and analyzed with Fragstats using another small C++ code. Simulations lasted 2.5 days over 250 cores (meaning that on a single core it would have taken 1.7 years). Prior to filtering, 12.6 billion landscapes were produced, using 1.5 TB disk space.

## 3 Results (not occurring in the main text)

**Figures S3, S4 and S5** provide an overview of model versions 1, 2 and 3, respectively, using different colours to represent the effect of the most important parameter “patch generation parameter 3” (determining patch isometry). They demonstrate that, for all model versions a large number of landscapes cannot be matched in terms of the number of the number of patches and the average patch size. Moreover, only for only few metrics (LSI and cohesion) all landscapes are covered by the range of maps produced by Dinamica, and only in its Model version 3. These deviations explain the poor performance of these model versions.

**Figure S6** provides the results of a performance evaluation of all models based on a PCA approach (see main text), including model versions 5 and 6. The exceptionally poor performance of these versions likely indicates the effect of practical complexities with implementing this more complex model version. First, the starting maps produced were often below 95% forest cover, and abrupt changes in forest cover made it difficult to obtain maps for the required forest-cover increments (which required several calibration steps and a larger number of output maps). Second, the speed of expansion of roads versus patches seemed to strongly affect the results, and the moderating parameters were perhaps not controlled effectively enough – thus producing highly fragmented maps, of low realism. While we are certain that these complexities could be resolved with further adjustments and calibration, we see such efforts as exceeding beyond the scope of this paper.

## 4 Hints for implementation of Dinamica for landscape generation

For potential users of Dinamica who may wish to use it for generic purposes, we offer a documentation of the main activities required for implementation of the software. We further list the duration it required for the work (a total of 20 days for programming, 12 for simulation preparation, and 2.5 for actual simulation running over 250 processors). The actual model versions as designed with the GUI (Graphical User Interface) of Dinamica occur in **Figures S7**.

1. Gaining familiarity with the software and its capacities – included learning the GUI (2 days), experimenting with the built-in examples “game of life”, LUCC, and “patterns of change” (4 days), develop first own models and, in the process, understand Dinamica’s required inputs (e.g. uploading maps) and outputs, and gaining understanding of the parameters and outcomes of their values (4 days).
2. Developing model versions 1 and 2 based on the “pattern of change” example (3 days).
3. Operating the road constructor model based on the LUCC example: required identifying the relevant parameters and conducting first variations of these parameters to determine the exploration ranges for our simulations (5 days).
4. Building model versions 3 to 6, required understanding the relevant parameters (e.g. “weight of evidence”) and determining exploration ranges (5 days)
5. Building the C++ Program to change parameters in the Dinamica input files (5 days).
6. Developing the FRAGSTAT database writer, the program which read the parameter combination from the database, the batch files which controlled the whole simulation process and for avoiding bottlenecks and race conditions when using many cores in parallel: 5 days.
7. Testing and debugging: 2 days

The simulations themselves took about 2.5 days on 250 cores.

## 5 References

Grimm, V. et al. (2010) The ODD protocol: A review and first update. Ecological Modelling 221:2760-2768.

Grimm, V. et al. (2006) A standard protocol for describing individual-based and agent-based models. Ecological Modelling 198:115-126.

Soares, B.S., Rodrigues, H.R., Costa, W.L.S. 2009. Modeling Environmental Dynamics with Dinamica EGO. Centro de Sensoriamento Remoto/Universidade Federal de Minas Gerais. Belo Horizonte, Brazil. ISBN: 978-85-910119-0-2. <http://www.csr.ufmg.br/dinamica/tutorial/Dinamica_EGO_guidebook.pdf>

Soares, B., A. Alencar, D. Nepstad, G. Cerqueira, M. D. V. Diaz, S. Rivero, L. Solorzano, and E. Voll. (2004) Simulating the response of land-cover changes to road paving and governance along a major Amazon highway: the Santarem-Cuiaba corridor. Global Change Biology 10:745-764.

Soares, B.S., Cerqueira, G.C., Pennachin, C.L. (2002) Dinamica - a stochastic cellular automata model designed to simulate the landscape dynamics in an Amazonian colonization frontier. Ecological Modelling 154:217-235.

**Soares B.S.; Corradi Filho L.; Cerqueira G.C.; Araujo W.L. (2003)** [Simulating the spatial patterns of change through the use of the dinamica model](http://iris.sid.inpe.br:1908/rep/ltid.inpe.br/sbsr/2002/11.16.12.49/Cover?mirror=sid.inpe.br/banon/2001/03.14.09.08.12&metadatarepository=ltid.inpe.br/sbsr/2002/11.16.12.49.53)**. In: Simpósio Brasileiro de Sensoriamento Remoto (Anais XI SBSR), 11. Belo Horizonte, Brazil. Instituto Nacional de Pesquisas Espaciais: pp. 721-728.**

## 6 Tables

**Table S1**

1. **Processes included, the number of acting parameters explored in this study (# Param.), and how these were included in the different model versions. For convenience of replicating the model versions with Dinamica we provide the syntax for landscape generation.**

| **Process** | **# Param.** | **Dinamica Syntax** | **Model versions** | | | | |
| --- | --- | --- | --- | --- | --- | --- | --- |
|  |  |  | **1** | **2** | **3** | **4** | **5-6** |
| Transition | 1 | Transition matrix | + | + | + | + | + |
| Patch creation* | 3 | Functor: Patcher | + | + | + | + | + |
| Patch expansion | 4 | Functor: Expander | **-** | + | - | + | + |
| Road creation** | 4 | Functor: road constructor | - | - | + | + | + |
| Road creation in parallel | 2 | Functor: repeat | - | - | - | - | + |
| **Additional parameters and inputs** | | | | | | | |
| Transition prob. | 1 | Transition Matrix | + | + | + | + | + |
| Additional param. | 7 or 9*** | Weights of evidence | w | w | w+ | w+ | w+ |
| Input maps |  |  | 1 | 1 | 3 | 3 | 3 |

1. ** To activate patch creation processes go to the Dinamica function “Allocate Transitions”.*
2. *** Road creation was performed prior to patch creation and expansion.*
3. **** weights of evidence alone (marked “w”) accounts for 7 parameters (see table S3).*

**Table S2: Input parameter values and ranges**

The table lists the parameter values, and ranges if explored, for each of the four model versions.

| ***Model version:*** | ***1*** | ***2*** | ***3*** | ***4*** | ***5*** | ***6*** |
| --- | --- | --- | --- | --- | --- | --- |
| **Parameter name** | **Values** | | | | | |
| Transition Forest  to Matrix | 0.01 | | | | 0.003 | |
| ***Patche creation:*** | | | | | | |
| Mean patch size | 0.1, 0.5, 1.0, 2.5, 5.0, 10, 25, 50, 75 | 1.0, 5.0, 10, 25, 50, 75 | | | | |
| Patch size variance | 0.1, 0.5, 1.0, 2.5, 5.0, 10, 25, 50, 75 | 5** | | | | |
| Patch isometry | 0.1, 0.5, 1.0, 2.0, 5.0, 10, 100, 1000 | | | | 0.5, 1.0, 2.0, 5.0, 10, 100 | 0.1, 0.5, 1.0, 2.0, 5.0, 10, 100, 1000 |
| Per-cent transitions by expansion [%] | 0 | 0.25, 0.5, 0.75 | 0 | 0.25, 0.5, 0.75 | 0 | 0.5 |
| ***Patch expansion:*** | | | | | | |
| Mean patch size* | - | 1.0, 5.0, 10, 25, 50, 75 | - | 1.0, 5.0, 10, 25, 50, 75 | - | 1.0, 5.0, 10, 25, 50, 75 |
| Patch size variance* | - | 5** | - | 5** | - | 5** |
| Patch isometry* | 0.1, 0.5, 1.0, 2.0, 5.0, 10, 100, 1000 | | | | 0.5, 1.0, 2.0, 5.0, 10, 100 | 0.1, 0.5, 1.0, 2.0, 5.0, 10, 100, 1000 |
| ***Road constructor additional parameters:*** | | | | | | |
| Friction map parameter | 1  250 | | | | | |
| Attraction map parameter |
| ***Road constructor:*** | **-** | **-** | **Applied as input map** | | **Applied within model** | |
| Shorter distance to Roads for new segments | - | - | 10, 20, 30, 40, 70, 100 | | | |
| Longer distance to Roads for new segments | - | - | 3000 | | | |
| Number of horizontal quadrants | - | - | 10 | | | |
| Number of vertical quadrants | - | - | 10 | | | |
| Iterations of road constructor | - | - | 150 | | 3 | |
| Iterations until saving of first output | 6 | | | | 15 | |
| Total overall iterations equals saving events (# iterations) | 50 | | | | | |

*value is coupled to that of the same parameter of the *Patch creator* function.

**Parameters were fixed after a preliminary analysis indicating little impact of the parameter on model outcomes.

**Table S3: Weights of evidence utilised in the model versions 3 to 6.**

First line depicts distance ranges [m], second line depicts the probability for transition from forest (class 3) to open habitat (class 2).

| Distance class to road | 0:100 | 100:200 | 200:300 | 300:400 | 400:500 | 500:600 | 600:100000 |
| --- | --- | --- | --- | --- | --- | --- | --- |
| Transition probability | 10 | 7 | 5 | 2 | 1 | 0.5 | 0 |

**Table S4: Maps created per model version.**

First line provides the model version, second line the number of maps originally created (dividing then by 50, it shows the number of parameter combinations) and the third line delineates the number of maps used for analysis after dilution based on habitat cover criteria.

| Model version | 1 | 2 | 3 | 4 | 5 | 6 |
| --- | --- | --- | --- | --- | --- | --- |
| Maps created | 3,240,000 | 720,000 | 1,440,000 | 4,320,000 | 1,440,000 | 1,440,000 |
| Maps used | 1,231,200 | 297,600 | 552,000 | 1,623,800 | 450,500 | 451,200 |

## 7 Figures

**Figure S1**

1. Illustrative examples of the maps produced by the 4 model versions (from top to bottom), each with several examples along the simulation process (from left to right).


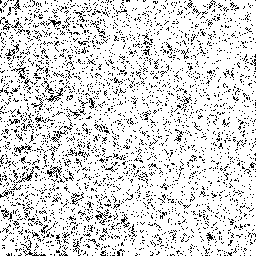

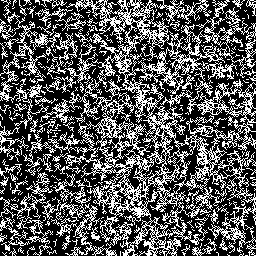

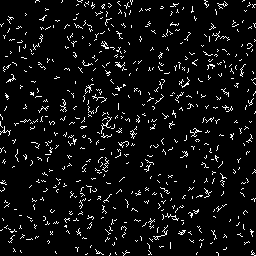

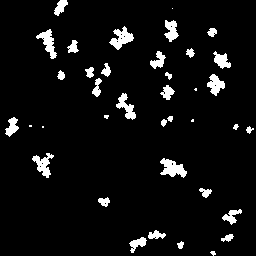

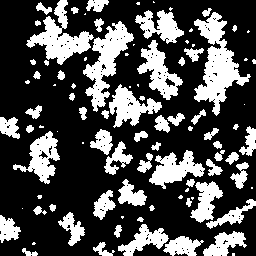

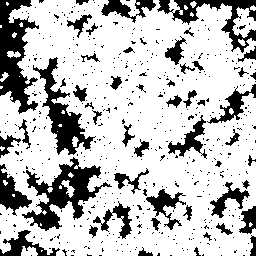

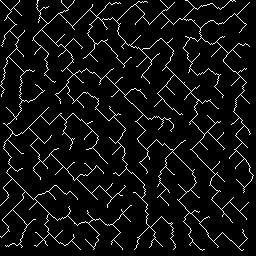

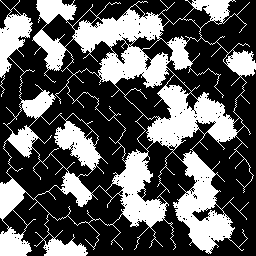

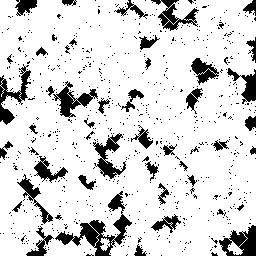

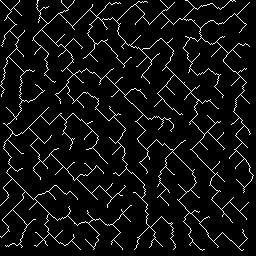

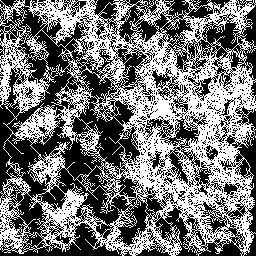

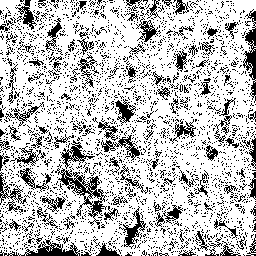


**Figure S2**

Illustrative example of outcomes of Model version 5 (first line) and 6 (second line), with iterative expansion of patches and roads to create a deforestation frontier (along the line from left to right).


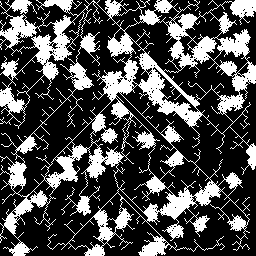

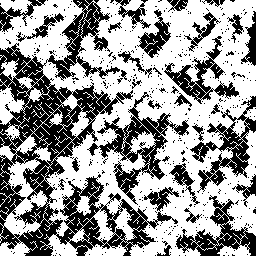

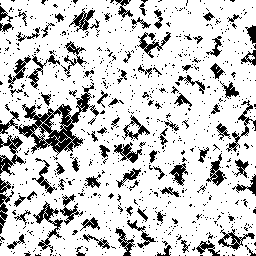


**
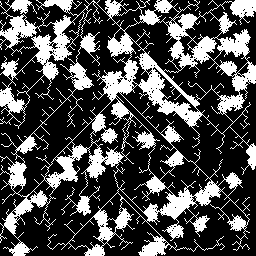

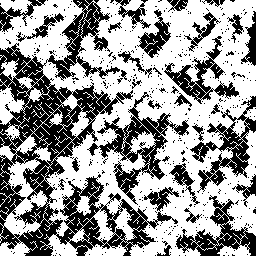

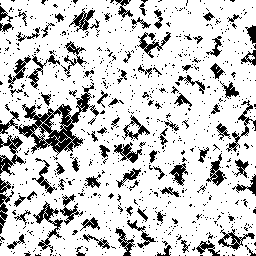
**


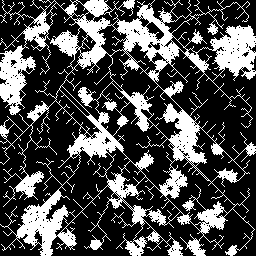

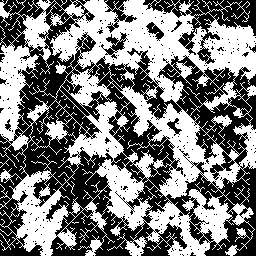

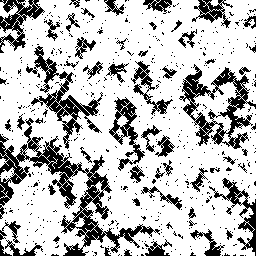


**Figure S3 (overview model version 1)**


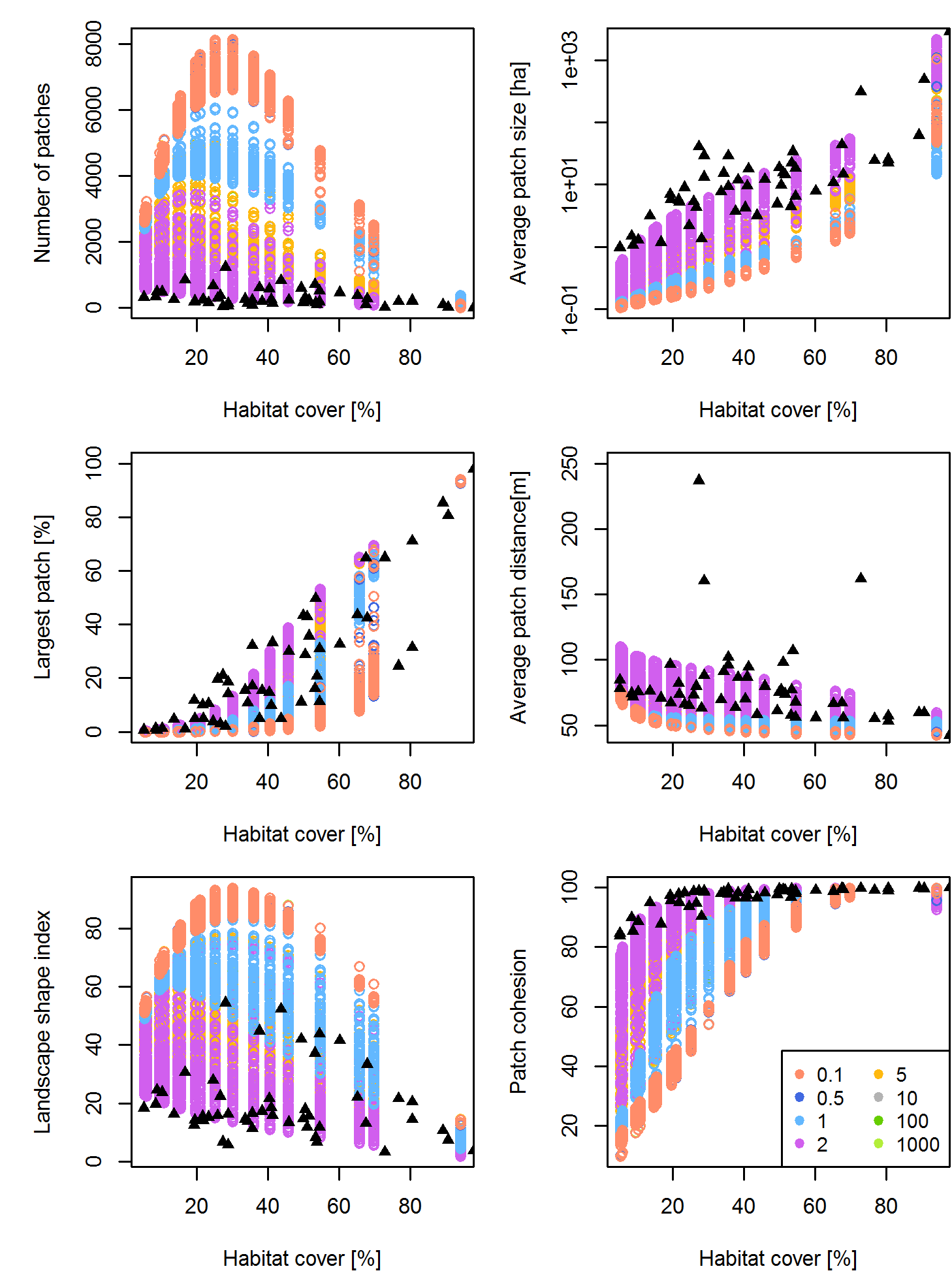


**Figure S4 (overview model version 2)**


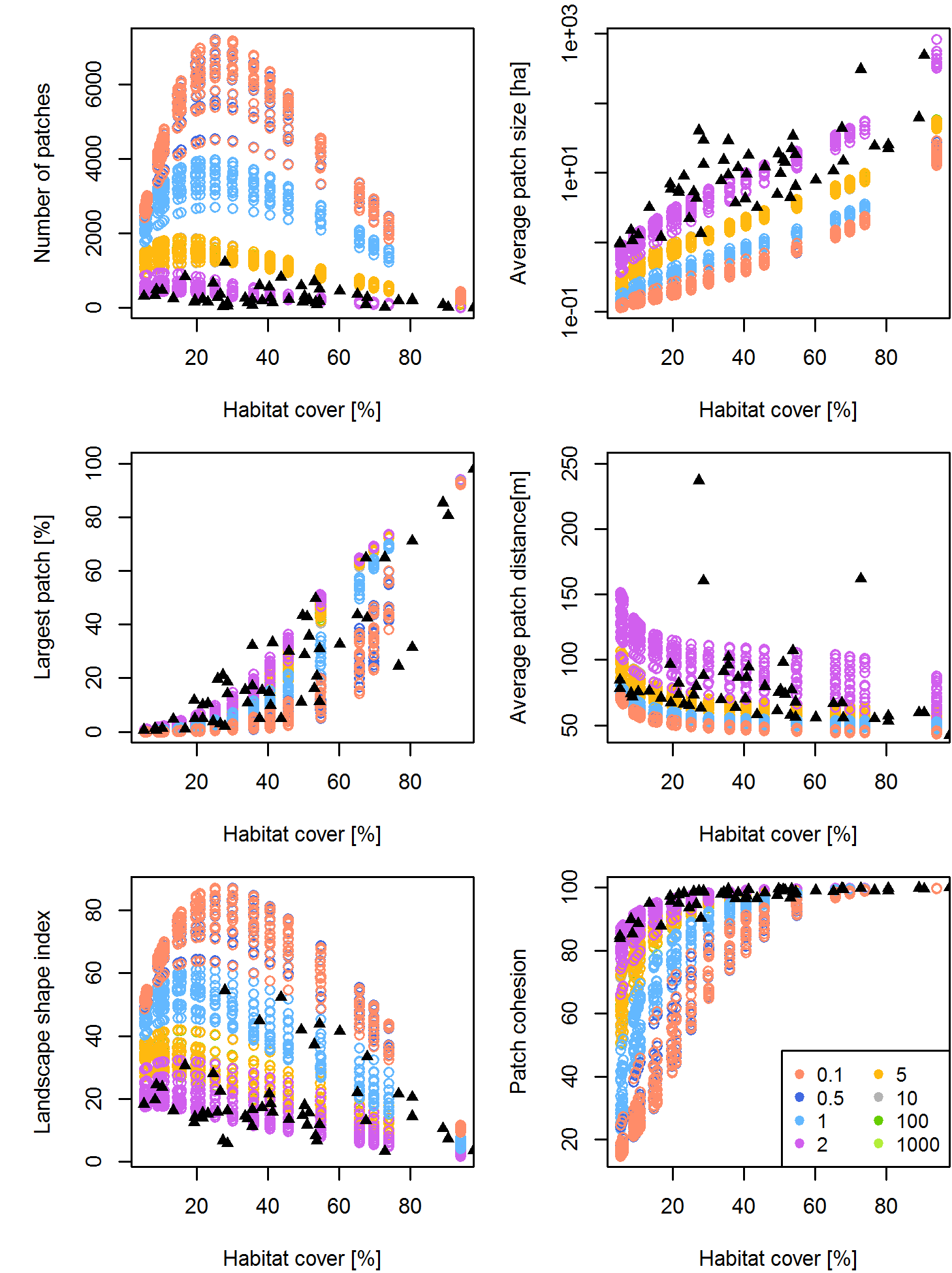


**Figure S5 (overview model version 3)**


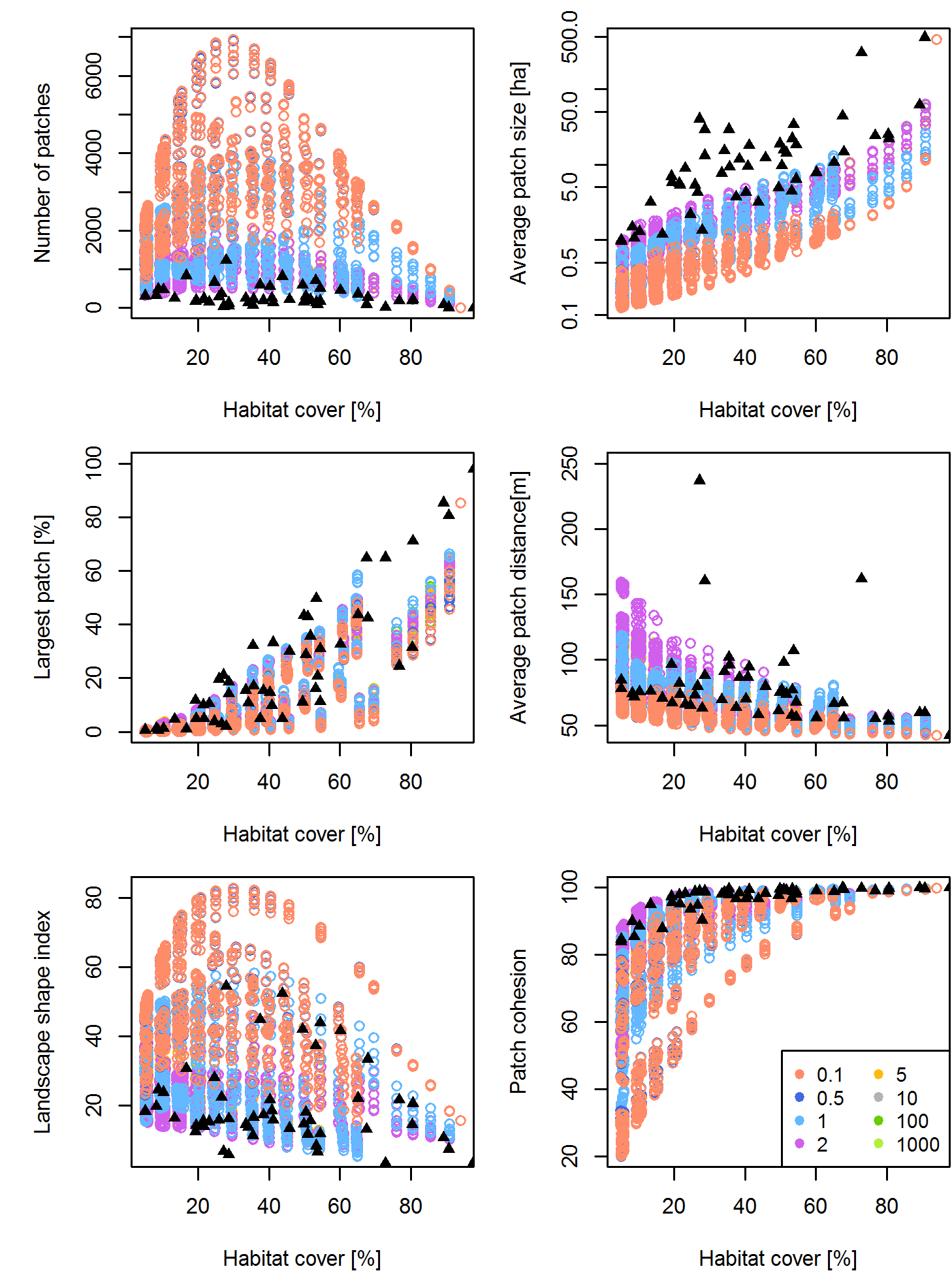


**Figure S6: Model performance (all models, all versions of Dinamica)**

1. Overall performance of all models, including Dinamica versions 1-6.


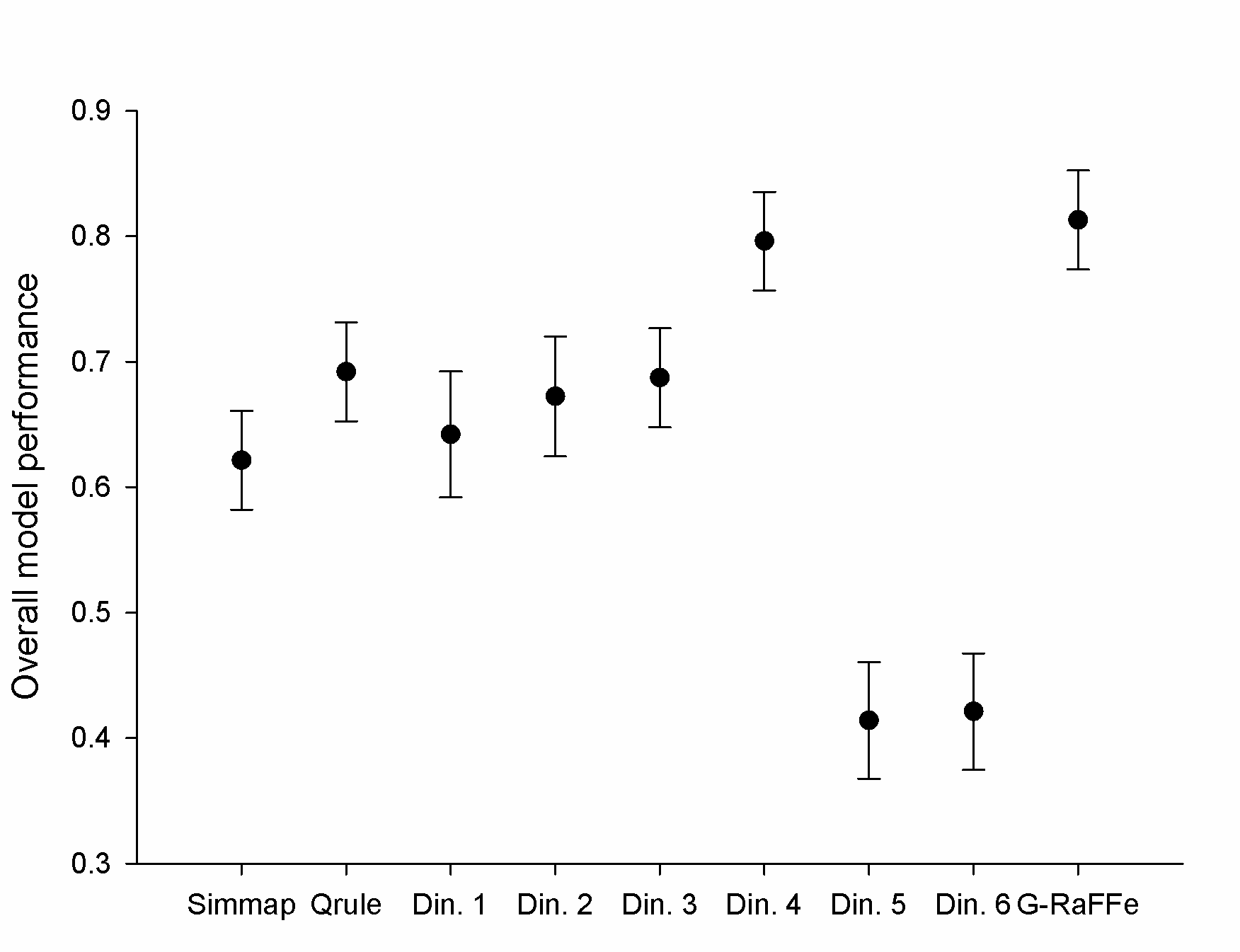


1.
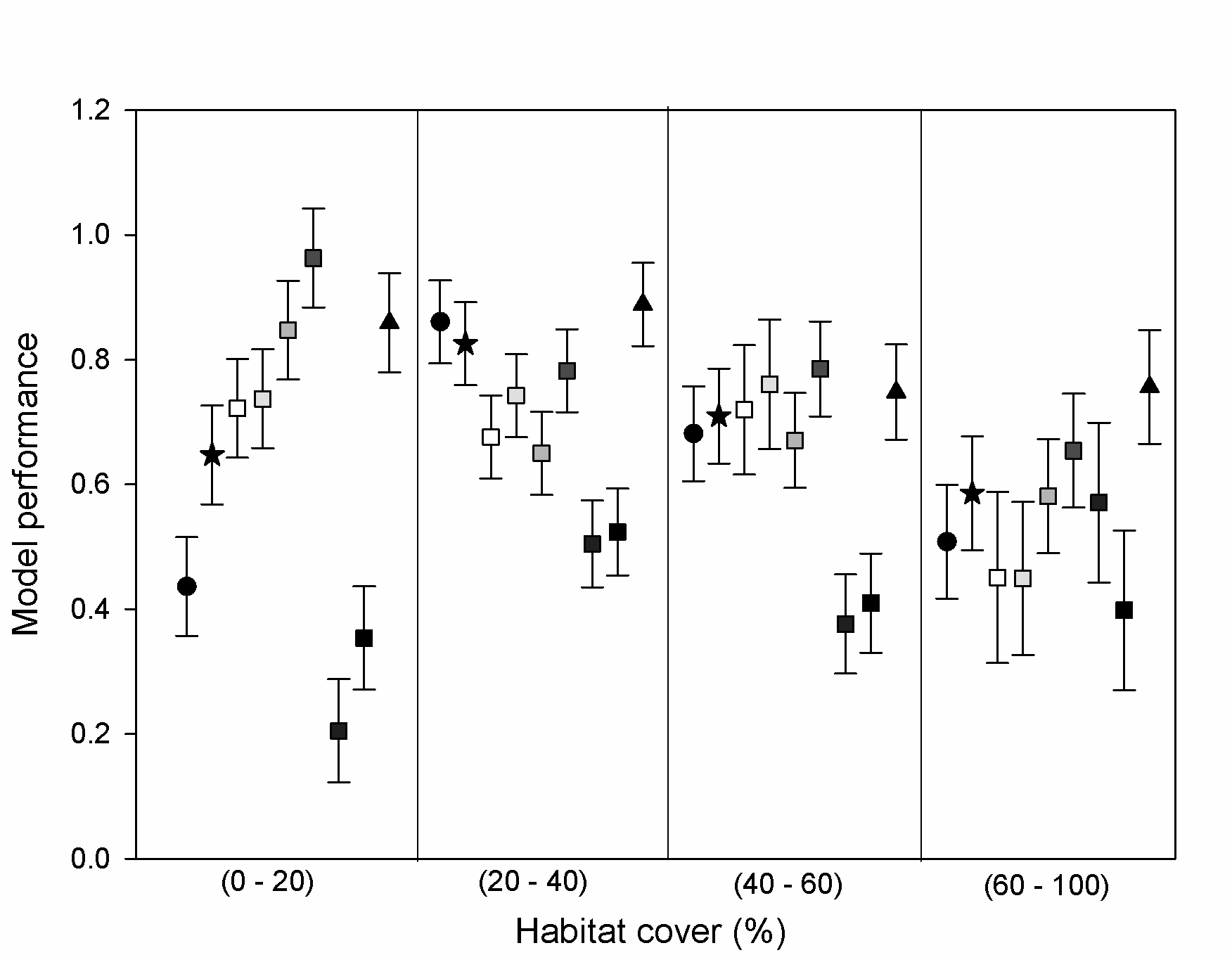
Same as (a) but separated to habitat cover categories

**Figure S7: Model design using Dinamica’s GUI**

1. Model version 1 and 2 (same design, but different parameters applied)


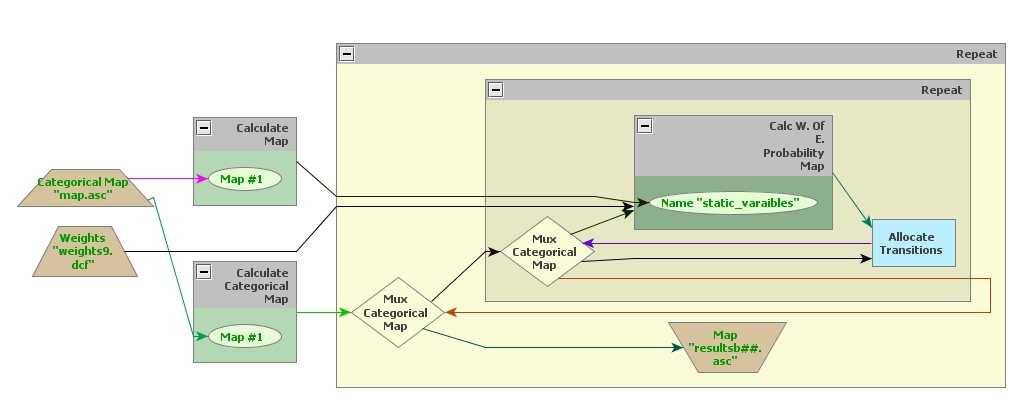


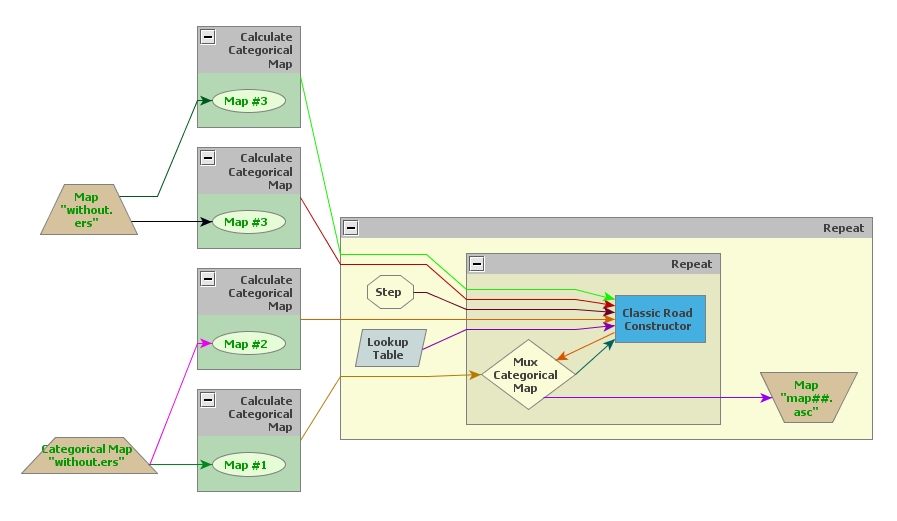


1. Road constructor for Model version 3 and 4
2. Model versions 3 and 4
3. Model versions 5 and 6 (same design, but different parameters applied)
