# Supplementary material for: Simple Process-Based Simulators for Generating Spatial Patterns of Habitat Loss and Fragmentation: A Review and Introduction to the G-RaFFe Model
Source: PLoS One. 2013 May 28;8(5):e64968. doi: 10.1371/journal.pone.0064968 (PMC3665680; doi:10.1371/journal.pone.0064968)
Supplement: Appendix S2 — Comparison of the attributes of the real and simulated landscapes for the landscape generators G-RaFFe, Qrule and Simmap. (DOCX) [file pone.0064968.s002.docx]

**Table S2: Comparison of the attributes of the real and simulated landscapes for the landscape generators *G-RaFFe*, *Qrule* and *Simmap*.**

Attributes presented in the following table include: Forest Cover (FC, in %); Number of patches, Largest Patch Index (LPI), Landscape Shape Index (LSI), Average patch size (Ha), Average Euclidean distance between patches (m), and patch cohesion. The fit represents the number of metrics which were simultaneously matched – i.e. real value falls within 95% intervals of the 100 landscapes generated for a given model parameter combination. Column (*) marks the selected landscapes occurring in **Figure 8** of the main text.

|  | ***** | **FC** | **Dominant land use** | **# Patches** | | **LPI** | | **LSI** | | **Ave Size** | | **Distance** | | **Cohesion** | | **Fits** |
| --- | --- | --- | --- | --- | --- | --- | --- | --- | --- | --- | --- | --- | --- | --- | --- | --- |
| **Source** |  |  |  | **Max** | **Min** | **Max** | **Min** | **Max** | **Min** | **Max** | **Min** | **Max** | **Min** | **Max** | **Min** |  |
| Real | a | 5.1 | Large farms | 313 | | 0.7 | | 18.4 | | 1.0 | | 84.6 | | 83.9 | |  |
| *G-RaFFe* |  | 5.0 |  | 389 | 89 | 2.1 | 0.2 | 21.3 | 9.0 | 3.3 | 0.8 | 86.8 | 58.6 | 93.3 | 75.5 | 6 |
| *Qrule* |  | 5.0 |  | 833 | 265 | 4.0 | 0.3 | 28.1 | 14.2 | 1.1 | 0.3 | 96.0 | 70.9 | 96.7 | 73.6 | 6 |
| *Simmap* |  | 5.0 |  | 317 | 236 | 0.7 | 0.1 | 17.7 | 15.2 | 1.4 | 0.7 | 217.7 | 162.4 | 84.2 | 74.2 | 4 |
| Real |  | 5.1 | Large farms | 307 | | 0.7 | | 18.3 | | 1.0 | | 78.1 | | 84.7 | |  |
| *G-RaFFe* |  | 5.0 |  | 389 | 89 | 2.1 | 0.2 | 21.3 | 9.0 | 3.3 | 0.8 | 86.8 | 58.6 | 93.3 | 75.5 | 6 |
| *Qrule* |  | 5.0 |  | 833 | 265 | 4.0 | 0.3 | 28.1 | 14.2 | 1.1 | 0.3 | 96.0 | 70.9 | 96.7 | 73.6 | 6 |
| *Simmap* |  | 5.0 |  | 10 | 1 | 100.0 | 0.0 | 4.0 | 1.0 | 5898.2 | 1.4 | 6520.2 | 0.0 | 100.0 | 75.3 | 3 |
| Real |  | 8.3 | Large farms | 330 | | 1.3 | | 19.7 | | 1.5 | | 74.2 | | 89.9 | |  |
| *G-RaFFe* |  | 10.0 |  | 638 | 124 | 4.5 | 0.4 | 26.4 | 10.6 | 4.8 | 0.9 | 94.3 | 61.0 | 94.6 | 78.1 | 6 |
| *Qrule* |  | 10.0 |  | 903 | 305 | 8.4 | 1.0 | 28.7 | 12.3 | 1.9 | 0.6 | 94.5 | 63.3 | 97.5 | 86.2 | 6 |
| *Simmap* |  | 10.0 |  | 344 | 267 | 2.9 | 0.4 | 17.8 | 15.0 | 2.9 | 1.2 | 190.1 | 150.4 | 93.7 | 83.9 | 4 |
| Real |  | 8.7 | Large farms | 485 | | 0.6 | | 24.5 | | 1.1 | | 71.8 | | 85.4 | |  |
| *G-RaFFe* |  | 10.0 |  | 638 | 124 | 4.5 | 0.4 | 26.4 | 10.6 | 4.8 | 0.9 | 94.3 | 61.0 | 94.6 | 78.1 | 6 |
| *Qrule* |  | 10.0 |  | 1104 | 464 | 7.2 | 0.6 | 32.1 | 17.4 | 1.3 | 0.5 | 87.5 | 64.6 | 97.2 | 84.7 | 6 |
| *Simmap* |  | 10.0 |  | 3 | 1 | 100.0 | 0.0 | 3.0 | 1.0 | 5898.2 | 2.9 | 7943.4 | 0.0 | 100.0 | 82.6 | 3 |
| Real | b | 10.3 | Large farms | 473 | | 1.3 | | 23.8 | | 1.3 | | 75.6 | | 88.5 | |  |
| *G-RaFFe* |  | 10.0 |  | 638 | 124 | 4.5 | 0.4 | 26.4 | 10.6 | 4.8 | 0.9 | 94.3 | 61.0 | 94.6 | 78.1 | 6 |
| *Qrule* |  | 10.0 |  | 903 | 305 | 8.4 | 1.0 | 28.7 | 12.3 | 1.9 | 0.6 | 94.5 | 63.3 | 97.5 | 86.2 | 6 |
| *Simmap* |  | 10.0 |  | 3 | 1 | 100.0 | 0.0 | 3.0 | 1.0 | 5898.2 | 2.9 | 7943.4 | 0.0 | 100.0 | 82.6 | 3 |
| Real |  | 13.4 | Large farms | 252 | | 4.7 | | 16.3 | | 3.1 | | 76.1 | | 95.0 | |  |
| *G-RaFFe* |  | 15.0 |  | 377 | 79 | 8.8 | 0.8 | 19.4 | 8.0 | 11.2 | 2.3 | 87.2 | 62.1 | 97.4 | 89.9 | 6 |
| *Qrule* |  | 15.0 |  | 971 | 243 | 13.6 | 1.8 | 28.7 | 10.6 | 3.6 | 0.9 | 86.3 | 63.2 | 98.6 | 92.2 | 6 |
| *Simmap* |  | 15.0 |  | 330 | 222 | 15.0 | 0.4 | 16.4 | 13.2 | 5.8 | 1.1 | 191.9 | 152.2 | 98.4 | 82.4 | 5 |
| Real |  | 16.7 | Large farms | 835 | | 1.0 | | 30.6 | | 1.2 | | 70.8 | | 87.8 | |  |
| *G-RaFFe* |  | 15.0 |  | 1321 | 418 | 2.4 | 0.3 | 36.1 | 20.0 | 2.1 | 0.7 | 74.9 | 55.2 | 89.6 | 74.6 | 6 |
| *Qrule* |  | 15.0 |  | 1489 | 523 | 10.8 | 1.1 | 38.0 | 18.5 | 1.7 | 0.6 | 78.6 | 62.6 | 97.2 | 86.6 | 5 |
| *Simmap* |  | 15.0 |  | 8 | 1 | 100.0 | 0.0 | 5.1 | 1.0 | 5898.2 | 2.9 | 8154.8 | 0.0 | 100.0 | 82.6 | 3 |
| Real | c | 19.2 | Large farms | 164 | | 11.8 | | 12.5 | | 6.9 | | 96.6 | | 97.3 | |  |
| *G-RaFFe* |  | 20.0 |  | 217 | 31 | 19.4 | 3.0 | 13.8 | 4.3 | 38.1 | 5.4 | 133.0 | 73.7 | 99.2 | 94.9 | 6 |
| *Qrule* |  | 20.0 |  | 419 | 66 | 19.6 | 4.8 | 15.3 | 4.3 | 17.9 | 2.8 | 143.6 | 58.6 | 99.2 | 96.6 | 6 |
| *Simmap* |  | 20.0 |  | 187 | 103 | 20.8 | 0.9 | 15.6 | 12.2 | 16.8 | 3.6 | 219.2 | 161.5 | 98.9 | 90.2 | 5 |
| Real |  | 19.4 | Large farms | 197 | | 4.9 | | 14.2 | | 5.8 | | 67.1 | | 95.6 | |  |
| *G-RaFFe* |  | 20.0 |  | 499 | 99 | 11.5 | 1.1 | 21.9 | 8.4 | 11.9 | 2.4 | 84.1 | 62.7 | 97.7 | 90.5 | 6 |
| *Qrule* |  | 20.0 |  | 611 | 147 | 19.2 | 3.8 | 20.0 | 6.4 | 8.0 | 1.9 | 90.0 | 62.0 | 99.2 | 95.4 | 6 |
| *Simmap* |  | 20.0 |  | 241 | 135 | 19.3 | 0.9 | 16.6 | 13.4 | 13.2 | 2.9 | 202.7 | 150.7 | 98.7 | 89.0 | 5 |
| Real |  | 21.6 | Large farms | 225 | | 10.0 | | 15.5 | | 5.7 | | 82.0 | | 97.8 | |  |
| *G-RaFFe* |  | 20.0 |  | 303 | 33 | 19.5 | 2.7 | 15.8 | 3.3 | 35.7 | 3.9 | 103.7 | 71.8 | 99.2 | 94.9 | 6 |
| *Qrule* |  | 20.0 |  | 695 | 204 | 18.3 | 4.9 | 20.9 | 7.9 | 5.7 | 1.7 | 102.5 | 58.0 | 99.0 | 96.0 | 6 |
| *Simmap* |  | 20.0 |  | 241 | 135 | 19.3 | 0.9 | 16.6 | 13.4 | 13.2 | 2.9 | 202.7 | 150.7 | 98.7 | 89.0 | 5 |
| Real |  | 21.7 | Large farms | 243 | | 4.9 | | 13.9 | | 5.3 | | 73.5 | | 95.0 | |  |
| *G-RaFFe* |  | 20.0 |  | 499 | 99 | 11.5 | 1.1 | 21.9 | 8.4 | 11.9 | 2.4 | 84.1 | 62.7 | 97.7 | 90.5 | 6 |
| *Qrule* |  | 20.0 |  | 695 | 204 | 18.3 | 4.9 | 20.9 | 7.9 | 5.7 | 1.7 | 102.5 | 58.0 | 99.0 | 96.0 | 5 |
| *Simmap* |  | 20.0 |  | 361 | 61 | 69.0 | 0.5 | 17.1 | 10.3 | 67.2 | 0.9 | 205.7 | 142.9 | 99.9 | 81.9 | 5 |
| Real |  | 23.2 | Large farms | 153 | | 10.6 | | 15.3 | | 8.9 | | 66.0 | | 98.2 | |  |
| *G-RaFFe* |  | 25.0 |  | 706 | 69 | 22.7 | 0.9 | 27.0 | 6.7 | 21.4 | 2.1 | 90.3 | 64.7 | 99.1 | 88.8 | 6 |
| *Qrule* |  | 25.0 |  | 684 | 124 | 24.5 | 6.1 | 20.6 | 4.7 | 11.7 | 2.2 | 111.8 | 57.3 | 99.3 | 96.6 | 6 |
| *Simmap* |  | 25.0 |  | 269 | 151 | 24.7 | 1.8 | 16.9 | 14.0 | 13.8 | 3.8 | 179.5 | 143.3 | 99.1 | 92.8 | 5 |
| Real |  | 24.6 | Large farms | 666 | | 3.9 | | 28.0 | | 2.2 | | 65.1 | | 93.6 | |  |
| *G-RaFFe* |  | 25.0 |  | 1015 | 251 | 8.3 | 0.7 | 30.1 | 13.7 | 5.9 | 1.5 | 76.1 | 58.8 | 96.1 | 86.1 | 6 |
| *Qrule* |  | 25.0 |  | 1453 | 480 | 23.0 | 2.5 | 36.0 | 15.0 | 3.1 | 1.0 | 83.6 | 59.3 | 99.0 | 93.5 | 6 |
| *Simmap* |  | 25.0 |  | 755 | 622 | 2.1 | 0.5 | 29.9 | 27.5 | 2.5 | 1.7 | 95.9 | 85.8 | 90.5 | 86.2 | 3 |
| Real |  | 25.8 | Large farms | 285 | | 19.6 | | 15.8 | | 5.3 | | 73.2 | | 98.7 | |  |
| *G-RaFFe* |  | 25.0 |  | 706 | 69 | 22.7 | 0.9 | 27.0 | 6.7 | 21.4 | 2.1 | 90.3 | 64.7 | 99.1 | 88.8 | 6 |
| *Qrule* |  | 25.0 |  | 1010 | 217 | 24.1 | 4.8 | 28.6 | 8.9 | 6.8 | 1.5 | 82.5 | 60.0 | 99.2 | 96.0 | 6 |
| *Simmap* |  | 25.0 |  | 425 | 256 | 23.2 | 1.9 | 18.5 | 15.3 | 8.5 | 1.8 | 153.6 | 127.1 | 98.8 | 90.5 | 5 |
| Real | d | 26.6 | Tree plantations | 369 | | 3.0 | | 22.4 | | 4.2 | | 79.7 | | 94.7 | |  |
| *G-RaFFe* |  | 25.0 |  | 706 | 69 | 22.7 | 0.9 | 27.0 | 6.7 | 21.4 | 2.1 | 90.3 | 64.7 | 99.1 | 88.8 | 6 |
| *Qrule* |  | 25.0 |  | 1453 | 480 | 23.0 | 2.5 | 36.0 | 15.0 | 3.1 | 1.0 | 83.6 | 59.3 | 99.0 | 93.5 | 4 |
| *Simmap* |  | 25.0 |  | 507 | 357 | 7.6 | 1.0 | 22.8 | 20.3 | 5.3 | 2.3 | 120.2 | 104.7 | 96.4 | 89.7 | 5 |
| Real |  | 27.3 | Large farms | 40 | | 21.1 | | 6.7 | | 40.2 | | 236.9 | | 99.0 | |  |
| *G-RaFFe* |  | 25.0 |  | 62 | 8 | 25.0 | 7.4 | 6.9 | 2.5 | 184.3 | 23.8 | 275.1 | 85.2 | 99.6 | 97.8 | 6 |
| *Qrule* |  | 25.0 |  | 242 | 28 | 25.1 | 9.7 | 10.1 | 2.7 | 52.7 | 6.1 | 268.4 | 61.7 | 99.5 | 97.7 | 6 |
| *Simmap* |  | 25.0 |  | 65 | 7 | 69.8 | 0.9 | 10.2 | 6.6 | 591.5 | 6.8 | 543.5 | 154.8 | 99.9 | 91.3 | 6 |
| Real | e | 27.9 | Small farms | 1229 | | 2.1 | | 54.5 | | 1.3 | | 63.1 | | 90.3 | |  |
| *G-RaFFe* |  | 30.0 |  | 1595 | 640 | 7.0 | 0.6 | 37.5 | 22.3 | 2.8 | 1.1 | 69.5 | 57.5 | 92.8 | 83.0 | 5 |
| *Qrule* |  | 30.0 |  | 1945 | 1063 | 22.5 | 3.7 | 43.4 | 25.3 | 1.7 | 0.9 | 66.8 | 56.4 | 98.7 | 93.8 | 3 |
| *Simmap* |  | 30.0 |  | 1258 | 1089 | 0.8 | 0.3 | 42.2 | 40.4 | 1.6 | 1.3 | 67.4 | 62.1 | 86.6 | 83.1 | 3 |
| Real |  | 28.7 | Tree plantations | 58 | | 18.6 | | 5.8 | | 29.2 | | 160.2 | | 98.6 | |  |
| *G-RaFFe* |  | 30.0 |  | 84 | 6 | 30.0 | 9.4 | 6.5 | 2.3 | 294.8 | 21.1 | 288.8 | 86.6 | 99.7 | 97.9 | 6 |
| *Qrule* |  | 30.0 |  | 198 | 17 | 30.1 | 10.6 | 9.3 | 1.8 | 104.1 | 8.9 | 164.0 | 60.7 | 99.6 | 98.2 | 6 |
| *Simmap* |  | 30.0 |  | 62 | 22 | 39.6 | 1.8 | 12.8 | 9.5 | 120.6 | 14.8 | 328.7 | 153.9 | 99.6 | 94.2 | 5 |
| Real |  | 28.7 | Small farms | 129 | | 14.2 | | 16.2 | | 13.1 | | 88.0 | | 99.0 | |  |
| *G-RaFFe* |  | 30.0 |  | 335 | 37 | 26.7 | 3.0 | 16.4 | 4.1 | 47.8 | 5.3 | 144.5 | 84.9 | 99.2 | 95.0 | 6 |
| *Qrule* |  | 30.0 |  | 563 | 114 | 29.5 | 8.4 | 17.2 | 5.6 | 15.5 | 3.1 | 95.2 | 58.9 | 99.4 | 97.1 | 6 |
| *Simmap* |  | 30.0 |  | 245 | 28 | 75.6 | 0.6 | 16.4 | 8.5 | 160.4 | 1.7 | 209.5 | 129.8 | 99.9 | 84.3 | 5 |
| Real |  | 33.5 | Large farms | 258 | | 15.5 | | 14.6 | | 7.7 | | 69.6 | | 98.0 | |  |
| *G-RaFFe* |  | 35.0 |  | 606 | 102 | 28.4 | 1.6 | 22.7 | 7.0 | 20.2 | 3.4 | 91.9 | 65.5 | 99.0 | 92.7 | 6 |
| *Qrule* |  | 35.0 |  | 416 | 66 | 34.6 | 12.3 | 13.9 | 4.0 | 31.4 | 5.0 | 149.2 | 59.2 | 99.6 | 97.8 | 5 |
| *Simmap* |  | 35.0 |  | 146 | 65 | 36.3 | 2.9 | 15.9 | 12.1 | 42.9 | 8.6 | 188.1 | 128.0 | 99.4 | 95.2 | 3 |
| Real |  | 34.2 | Tree plantations | 133 | | 10.7 | | 13.7 | | 15.2 | | 91.2 | | 98.5 | |  |
| *G-RaFFe* |  | 35.0 |  | 606 | 102 | 28.4 | 1.6 | 22.7 | 7.0 | 20.2 | 3.4 | 91.9 | 65.5 | 99.0 | 92.7 | 6 |
| *Qrule* |  | 35.0 |  | 416 | 66 | 34.6 | 12.3 | 13.9 | 4.0 | 31.4 | 5.0 | 149.2 | 59.2 | 99.6 | 97.8 | 5 |
| *Simmap* |  | 35.0 |  | 146 | 65 | 36.3 | 2.9 | 15.9 | 12.1 | 42.9 | 8.6 | 188.1 | 128.0 | 99.4 | 95.2 | 5 |
| Real |  | 35.5 | Tree plantations | 72 | | 32.1 | | 11.3 | | 29.1 | | 102.0 | | 99.5 | |  |
| *G-RaFFe* |  | 35.0 |  | 491 | 55 | 34.2 | 3.8 | 16.6 | 4.0 | 37.5 | 4.2 | 105.5 | 73.5 | 99.6 | 95.8 | 6 |
| *Qrule* |  | 35.0 |  | 416 | 66 | 34.6 | 12.3 | 13.9 | 4.0 | 31.4 | 5.0 | 149.2 | 59.2 | 99.6 | 97.8 | 6 |
| *Simmap* |  | 35.0 |  | 467 | 39 | 79.1 | 0.8 | 19.2 | 9.9 | 119.9 | 1.1 | 157.6 | 105.9 | 99.9 | 83.9 | 5 |
| Real |  | 35.6 | Tree plantations | 226 | | 17.1 | | 16.6 | | 9.3 | | 96.1 | | 97.8 | |  |
| *G-RaFFe* |  | 35.0 |  | 510 | 101 | 26.5 | 2.5 | 22.3 | 7.5 | 20.4 | 4.0 | 119.9 | 62.5 | 98.7 | 92.5 | 6 |
| *Qrule* |  | 35.0 |  | 949 | 183 | 33.6 | 7.8 | 26.0 | 6.3 | 11.2 | 2.2 | 81.9 | 60.1 | 99.5 | 97.1 | 5 |
| *Simmap* |  | 35.0 |  | 275 | 121 | 38.2 | 2.3 | 18.1 | 14.5 | 23.8 | 5.0 | 160.4 | 122.7 | 99.4 | 93.8 | 5 |
| Real |  | 37.6 | Tree plantations | 600 | | 5.0 | | 44.9 | | 3.7 | | 63.5 | | 96.6 | |  |
| *G-RaFFe* |  | 40.0 |  | 893 | 337 | 20.1 | 2.1 | 29.1 | 14.9 | 7.0 | 2.6 | 76.0 | 59.3 | 96.9 | 90.4 | 5 |
| *Qrule* |  | 40.0 |  | 2500 | 1729 | 30.3 | 4.8 | 53.3 | 39.3 | 1.4 | 0.9 | 57.1 | 52.0 | 98.9 | 94.9 | 3 |
| *Simmap* |  | 40.0 |  | 294 | 17 | 81.2 | 0.8 | 17.5 | 8.6 | 283.1 | 2.0 | 192.9 | 116.4 | 99.9 | 86.4 | 3 |
| Real |  | 38.4 | Tree plantations | 192 | | 15.4 | | 17.2 | | 11.8 | | 86.6 | | 98.3 | |  |
| *G-RaFFe* |  | 40.0 |  | 590 | 106 | 28.5 | 2.2 | 22.6 | 6.2 | 22.3 | 4.0 | 87.2 | 62.5 | 98.6 | 93.4 | 6 |
| *Qrule* |  | 40.0 |  | 702 | 175 | 39.1 | 13.5 | 20.7 | 6.9 | 13.5 | 3.4 | 76.9 | 59.3 | 99.5 | 98.0 | 5 |
| *Simmap* |  | 40.0 |  | 264 | 140 | 39.5 | 3.5 | 17.8 | 14.0 | 20.4 | 6.3 | 144.9 | 117.4 | 99.5 | 95.0 | 5 |
| Real |  | 40.3 | Tree plantations | 562 | | 14.6 | | 21.6 | | 4.2 | | 70.2 | | 96.6 | |  |
| *G-RaFFe* |  | 40.0 |  | 590 | 106 | 28.5 | 2.2 | 22.6 | 6.2 | 22.3 | 4.0 | 87.2 | 62.5 | 98.6 | 93.4 | 6 |
| *Qrule* |  | 40.0 |  | 1008 | 394 | 37.5 | 9.9 | 26.0 | 12.1 | 6.0 | 2.3 | 71.5 | 58.4 | 99.4 | 97.5 | 5 |
| *Simmap* |  | 40.0 |  | 567 | 372 | 10.3 | 2.0 | 30.6 | 28.0 | 6.9 | 3.8 | 82.5 | 70.5 | 97.4 | 93.8 | 3 |
| Real |  | 40.8 | Tree plantations | 253 | | 9.8 | | 18.5 | | 9.5 | | 86.8 | | 97.4 | |  |
| *G-RaFFe* |  | 40.0 |  | 590 | 106 | 28.5 | 2.2 | 22.6 | 6.2 | 22.3 | 4.0 | 87.2 | 62.5 | 98.6 | 93.4 | 6 |
| *Qrule* |  | 40.0 |  | 768 | 227 | 38.8 | 13.7 | 21.4 | 8.5 | 10.4 | 3.1 | 85.5 | 57.8 | 99.5 | 98.0 | 3 |
| *Simmap* |  | 40.0 |  | 314 | 209 | 12.1 | 1.9 | 28.1 | 26.2 | 12.2 | 7.0 | 92.2 | 75.8 | 97.8 | 94.2 | 5 |
| Real |  | 41.3 | Tree plantations | 136 | | 33.3 | | 15.8 | | 17.9 | | 94.5 | | 99.4 | |  |
| *G-RaFFe* |  | 40.0 |  | 534 | 44 | 39.6 | 4.0 | 18.8 | 3.5 | 53.6 | 4.4 | 102.3 | 72.4 | 99.6 | 95.7 | 6 |
| *Qrule* |  | 40.0 |  | 446 | 70 | 39.9 | 13.9 | 16.1 | 3.3 | 33.5 | 5.3 | 114.4 | 58.2 | 99.6 | 98.2 | 6 |
| *Simmap* |  | 40.0 |  | 147 | 51 | 39.9 | 3.4 | 16.5 | 12.1 | 59.4 | 11.0 | 175.6 | 117.4 | 99.5 | 95.6 | 5 |
| Real |  | 43.7 | Small farms | 816 | | 4.9 | | 52.3 | | 3.2 | | 58.0 | | 96.6 | |  |
| *G-RaFFe* |  | 45.0 |  | 854 | 183 | 27.0 | 1.7 | 25.6 | 8.5 | 14.5 | 3.1 | 63.3 | 51.2 | 98.5 | 93.0 | 5 |
| *Qrule* |  | 45.0 |  | 1514 | 764 | 40.4 | 11.3 | 36.9 | 20.8 | 3.5 | 1.7 | 62.7 | 55.2 | 99.4 | 97.5 | 3 |
| *Simmap* |  | 45.0 |  | 898 | 728 | 2.1 | 0.7 | 52.8 | 50.6 | 3.5 | 2.7 | 49.8 | 46.8 | 91.6 | 89.0 | 3 |
| Real |  | 45.8 | Tree plantations | 222 | | 29.9 | | 13.4 | | 12.2 | | 79.8 | | 98.3 | |  |
| *G-RaFFe* |  | 45.0 |  | 483 | 69 | 37.0 | 4.0 | 19.3 | 5.0 | 38.5 | 5.5 | 82.6 | 53.2 | 99.1 | 94.7 | 6 |
| *Qrule* |  | 45.0 |  | 785 | 184 | 44.1 | 18.1 | 21.9 | 7.1 | 14.3 | 3.4 | 82.6 | 57.4 | 99.6 | 98.2 | 6 |
| *Simmap* |  | 45.0 |  | 513 | 19 | 85.4 | 1.5 | 19.1 | 8.6 | 265.4 | 1.6 | 146.2 | 79.1 | 100.0 | 87.9 | 6 |
| Real |  | 49.4 | Small farms | 594 | | 11.2 | | 42.0 | | 4.9 | | 61.0 | | 97.6 | |  |
| *G-RaFFe* |  | 50.0 |  | 711 | 98 | 49.3 | 10.8 | 17.4 | 4.2 | 30.1 | 4.1 | 64.9 | 55.5 | 99.7 | 97.1 | 5 |
| *Qrule* |  | 50.0 |  | 1261 | 546 | 47.8 | 11.9 | 31.3 | 12.1 | 5.4 | 2.3 | 67.5 | 54.9 | 99.6 | 97.5 | 4 |
| *Simmap* |  | 50.0 |  | 624 | 436 | 28.6 | 2.7 | 45.8 | 43.5 | 6.8 | 4.6 | 53.0 | 49.2 | 99.1 | 95.1 | 4 |
| Real |  | 49.8 | Tree plantations | 156 | | 43.4 | | 14.7 | | 18.8 | | 75.2 | | 99.6 | |  |
| *G-RaFFe* |  | 50.0 |  | 435 | 51 | 48.9 | 7.9 | 16.4 | 3.8 | 57.8 | 6.8 | 100.8 | 73.0 | 99.6 | 97.0 | 6 |
| *Qrule* |  | 50.0 |  | 592 | 110 | 49.9 | 17.3 | 16.9 | 3.9 | 27.0 | 5.0 | 76.7 | 57.1 | 99.7 | 98.5 | 6 |
| *Simmap* |  | 50.0 |  | 241 | 139 | 43.7 | 6.8 | 27.5 | 24.6 | 22.3 | 11.7 | 82.8 | 71.9 | 99.6 | 97.7 | 5 |
| Real |  | 50.3 | Tree plantations | 306 | | 28.8 | | 18.0 | | 9.7 | | 77.2 | | 98.9 | |  |
| *G-RaFFe* |  | 50.0 |  | 473 | 98 | 42.4 | 3.9 | 19.1 | 6.3 | 30.1 | 6.2 | 97.6 | 64.9 | 99.2 | 94.9 | 6 |
| *Qrule* |  | 50.0 |  | 710 | 172 | 48.9 | 16.6 | 20.8 | 6.0 | 17.1 | 4.1 | 85.8 | 57.8 | 99.7 | 98.5 | 6 |
| *Simmap* |  | 50.0 |  | 374 | 238 | 43.4 | 8.1 | 28.3 | 26.2 | 13.0 | 7.5 | 77.3 | 68.1 | 99.6 | 97.5 | 5 |
| Real |  | 51.0 | Large farms | 194 | | 42.9 | | 11.7 | | 15.5 | | 98.0 | | 99.3 | |  |
| *G-RaFFe* |  | 50.0 |  | 318 | 45 | 49.3 | 8.8 | 12.9 | 3.0 | 65.5 | 9.3 | 104.5 | 72.7 | 99.7 | 97.5 | 6 |
| *Qrule* |  | 50.0 |  | 710 | 172 | 48.9 | 16.6 | 20.8 | 6.0 | 17.1 | 4.1 | 85.8 | 57.8 | 99.7 | 98.5 | 5 |
| *Simmap* |  | 50.0 |  | 624 | 436 | 28.6 | 2.7 | 45.8 | 43.5 | 6.8 | 4.6 | 53.0 | 49.2 | 99.1 | 95.1 | 0 |
| Real | f | 51.6 | Tree plantations | 214 | | 35.7 | | 15.6 | | 14.2 | | 73.8 | | 99.2 | |  |
| *G-RaFFe* |  | 50.0 |  | 473 | 98 | 42.4 | 3.9 | 19.1 | 6.3 | 30.1 | 6.2 | 97.6 | 64.9 | 99.2 | 94.9 | 6 |
| *Qrule* |  | 50.0 |  | 710 | 172 | 48.9 | 16.6 | 20.8 | 6.0 | 17.1 | 4.1 | 85.8 | 57.8 | 99.7 | 98.5 | 6 |
| *Simmap* |  | 50.0 |  | 241 | 139 | 43.7 | 6.8 | 27.5 | 24.6 | 22.3 | 11.7 | 82.8 | 71.9 | 99.6 | 97.7 | 5 |
| Real |  | 53.1 | Tree plantations | 708 | | 16.2 | | 37.3 | | 4.4 | | 57.7 | | 96.7 | |  |
| *G-RaFFe* |  | 55.0 |  | 774 | 161 | 35.5 | 3.4 | 23.8 | 8.0 | 20.1 | 4.2 | 59.4 | 50.2 | 99.2 | 94.5 | 5 |
| *Qrule* |  | 55.0 |  | 1187 | 659 | 51.3 | 23.6 | 31.2 | 16.2 | 4.9 | 2.7 | 66.6 | 54.3 | 99.6 | 98.3 | 3 |
| *Simmap* |  | 55.0 |  | 479 | 24 | 87.3 | 3.1 | 18.0 | 8.1 | 215.0 | 2.7 | 138.7 | 104.3 | 100.0 | 93.7 | 3 |
| Real |  | 53.4 | Tree plantations | 143 | | 49.7 | | 8.3 | | 22.0 | | 77.0 | | 99.5 | |  |
| *G-RaFFe* |  | 55.0 |  | 219 | 17 | 54.9 | 9.7 | 11.1 | 1.7 | 190.8 | 14.8 | 113.7 | 49.9 | 99.8 | 97.7 | 6 |
| *Qrule* |  | 55.0 |  | 556 | 121 | 54.8 | 23.9 | 15.2 | 4.4 | 26.9 | 5.8 | 91.4 | 57.6 | 99.7 | 98.8 | 6 |
| *Simmap* |  | 55.0 |  | 219 | 8 | 85.1 | 2.3 | 16.0 | 6.5 | 614.7 | 4.7 | 190.9 | 70.0 | 100.0 | 92.6 | 6 |
| Real |  | 53.8 | Tree plantations | 94 | | 20.8 | | 6.6 | | 33.7 | | 107.0 | | 98.8 | |  |
| *G-RaFFe* |  | 55.0 |  | 219 | 17 | 54.9 | 9.7 | 11.1 | 1.7 | 190.8 | 14.8 | 113.7 | 49.9 | 99.8 | 97.7 | 6 |
| *Qrule* |  | 55.0 |  | 187 | 31 | 55.2 | 18.1 | 7.9 | 2.2 | 104.9 | 17.3 | 137.9 | 57.5 | 99.8 | 98.9 | 5 |
| *Simmap* |  | 55.0 |  | 219 | 8 | 85.1 | 2.3 | 16.0 | 6.5 | 614.7 | 4.7 | 190.9 | 70.0 | 100.0 | 92.6 | 6 |
| Real |  | 54.5 | Large farms | 177 | | 30.9 | | 11.8 | | 18.1 | | 67.9 | | 98.8 | |  |
| *G-RaFFe* |  | 55.0 |  | 414 | 55 | 47.9 | 3.8 | 19.5 | 4.7 | 59.0 | 7.8 | 91.4 | 60.9 | 99.5 | 95.8 | 6 |
| *Qrule* |  | 55.0 |  | 556 | 121 | 54.8 | 23.9 | 15.2 | 4.4 | 26.9 | 5.8 | 91.4 | 57.6 | 99.7 | 98.8 | 6 |
| *Simmap* |  | 55.0 |  | 206 | 54 | 66.1 | 12.7 | 15.9 | 11.5 | 74.2 | 12.0 | 132.0 | 101.5 | 99.9 | 98.2 | 5 |
| Real |  | 54.5 | Small farms | 502 | | 11.3 | | 43.8 | | 6.4 | | 56.0 | | 98.0 | |  |
| *G-RaFFe* |  | 55.0 |  | 532 | 88 | 41.5 | 4.9 | 16.8 | 5.4 | 36.9 | 6.1 | 66.0 | 52.8 | 99.1 | 95.9 | 5 |
| *Qrule* |  | 55.0 |  | 1093 | 490 | 52.7 | 16.4 | 26.3 | 12.1 | 6.6 | 3.0 | 66.0 | 54.1 | 99.6 | 98.6 | 3 |
| *Simmap* |  | 55.0 |  | 670 | 508 | 48.5 | 7.1 | 54.3 | 52.4 | 6.6 | 4.9 | 46.5 | 44.6 | 99.7 | 98.0 | 3 |
| Real |  | 60.1 | Small farms | 453 | | 32.7 | | 41.5 | | 7.8 | | 55.9 | | 99.1 | |  |
| *G-RaFFe* |  | 60.0 |  | 460 | 43 | 47.7 | 7.4 | 17.2 | 3.8 | 82.3 | 7.7 | 64.4 | 52.1 | 99.5 | 96.6 | 5 |
| *Qrule* |  | 60.0 |  | 820 | 285 | 58.7 | 21.2 | 22.1 | 9.2 | 12.4 | 4.3 | 66.6 | 54.3 | 99.7 | 98.8 | 5 |
| *Simmap* |  | 60.0 |  | 515 | 26 | 87.0 | 4.4 | 19.4 | 7.3 | 198.2 | 2.2 | 135.0 | 96.9 | 100.0 | 91.8 | 4 |
| Real | g | 65.1 | Tree plantations | 361 | | 43.6 | | 22.1 | | 10.6 | | 66.7 | | 98.8 | |  |
| *G-RaFFe* |  | 65.0 |  | 614 | 265 | 46.2 | 4.1 | 23.9 | 12.4 | 14.5 | 6.2 | 68.1 | 48.5 | 99.0 | 94.6 | 6 |
| *Qrule* |  | 65.0 |  | 320 | 71 | 65.0 | 28.9 | 10.6 | 3.2 | 54.2 | 12.0 | 110.3 | 55.7 | 99.8 | 99.4 | 2 |
| *Simmap* |  | 65.0 |  | 504 | 15 | 89.9 | 3.2 | 18.7 | 6.6 | 351.5 | 2.3 | 144.3 | 82.6 | 100.0 | 92.5 | 4 |
| Real |  | 67.4 | Tree plantations | 91 | | 64.8 | | 13.1 | | 43.7 | | 67.0 | | 99.8 | |  |
| *G-RaFFe* |  | 65.0 |  | 175 | 14 | 64.9 | 12.1 | 13.6 | 2.1 | 273.8 | 21.9 | 155.1 | 59.2 | 99.9 | 98.1 | 6 |
| *Qrule* |  | 65.0 |  | 320 | 71 | 65.0 | 28.9 | 10.6 | 3.2 | 54.2 | 12.0 | 110.3 | 55.7 | 99.8 | 99.4 | 5 |
| *Simmap* |  | 65.0 |  | 142 | 73 | 66.6 | 56.8 | 23.5 | 20.9 | 53.4 | 26.4 | 77.5 | 64.8 | 99.9 | 99.8 | 5 |
| Real |  | 67.9 | Small farms | 273 | | 42.4 | | 33.4 | | 14.7 | | 55.5 | | 99.5 | |  |
| *G-RaFFe* |  | 70.0 |  | 448 | 48 | 60.4 | 6.9 | 15.8 | 3.8 | 86.0 | 9.2 | 63.9 | 50.2 | 99.6 | 96.9 | 5 |
| *Qrule* |  | 70.0 |  | 445 | 125 | 69.8 | 36.6 | 14.1 | 3.9 | 33.1 | 9.3 | 74.9 | 55.1 | 99.8 | 99.3 | 5 |
| *Simmap* |  | 70.0 |  | 476 | 3 | 92.3 | 3.8 | 18.5 | 5.8 | 1815.6 | 3.3 | 140.6 | 42.4 | 100.0 | 94.3 | 5 |
| Real |  | 72.8 | Tree plantations | 14 | | 64.9 | | 3.3 | | 306.5 | | 161.9 | | 99.8 | |  |
| *G-RaFFe* |  | 75.0 |  | 69 | 5 | 75.0 | 30.0 | 4.5 | 1.4 | 884.7 | 64.1 | 227.6 | 64.0 | 99.9 | 99.3 | 6 |
| *Qrule* |  | 75.0 |  | 143 | 13 | 75.3 | 52.2 | 5.9 | 1.6 | 341.8 | 31.0 | 184.6 | 50.7 | 99.9 | 99.4 | 6 |
| *Simmap* |  | 75.0 |  | 90 | 1 | 96.1 | 5.8 | 12.4 | 2.6 | 5667.7 | 18.6 | 318.9 | 0.0 | 100.0 | 96.9 | 6 |
| Real |  | 76.7 | Tree plantations | 186 | | 24.5 | | 21.6 | | 24.3 | | 55.3 | | 99.1 | |  |
| *G-RaFFe* |  | 75.0 |  | 958 | 78 | 73.0 | 8.4 | 22.2 | 3.1 | 56.7 | 4.6 | 63.0 | 49.0 | 99.7 | 96.9 | 6 |
| *Qrule* |  | 75.0 |  | 453 | 167 | 74.4 | 64.2 | 15.1 | 5.5 | 26.5 | 9.8 | 71.4 | 54.1 | 99.8 | 99.4 | 3 |
| *Simmap* |  | 75.0 |  | 549 | 8 | 93.0 | 3.0 | 19.6 | 5.1 | 681.9 | 2.4 | 132.9 | 81.9 | 100.0 | 91.9 | 4 |
| Real |  | 80.5 | Small farms | 188 | | 31.5 | | 14.4 | | 25.2 | | 57.0 | | 99.0 | |  |
| *G-RaFFe* |  | 80.0 |  | 331 | 130 | 52.6 | 8.9 | 16.7 | 8.5 | 36.3 | 14.3 | 64.3 | 45.0 | 99.1 | 96.7 | 6 |
| *Qrule* |  | 80.0 |  | 460 | 160 | 79.6 | 71.0 | 15.6 | 5.9 | 29.5 | 10.3 | 73.2 | 52.4 | 99.9 | 99.6 | 4 |
| *Simmap* |  | 80.0 |  | 512 | 2 | 95.8 | 6.5 | 20.0 | 4.2 | 2825.9 | 2.9 | 144.0 | 78.9 | 100.0 | 94.5 | 5 |
| Real |  | 80.5 | Small farms | 216 | | 71.2 | | 20.6 | | 22.0 | | 53.2 | | 99.8 | |  |
| *G-RaFFe* |  | 80.0 |  | 331 | 58 | 79.2 | 14.0 | 12.4 | 4.3 | 81.4 | 14.3 | 68.4 | 48.9 | 99.9 | 98.1 | 5 |
| *Qrule* |  | 80.0 |  | 460 | 160 | 79.6 | 71.0 | 15.6 | 5.9 | 29.5 | 10.3 | 73.2 | 52.4 | 99.9 | 99.6 | 5 |
| *Simmap* |  | 80.0 |  | 253 | 3 | 95.3 | 9.2 | 14.3 | 3.6 | 1871.0 | 8.1 | 192.6 | 48.3 | 100.0 | 96.8 | 5 |
| Real |  | 89.2 | Small farms | 85 | | 85.3 | | 10.6 | | 61.9 | | 59.5 | | 99.9 | |  |
| *G-RaFFe* |  | 90.0 |  | 196 | 10 | 89.9 | 13.2 | 11.5 | 4.4 | 530.8 | 27.1 | 65.7 | 42.4 | 100.0 | 98.2 | 6 |
| *Qrule* |  | 90.0 |  | 156 | 39 | 90.1 | 83.3 | 5.3 | 2.0 | 136.6 | 34.0 | 77.5 | 51.1 | 99.9 | 99.7 | 5 |
| *Simmap* |  | 90.0 |  | 481 | 1 | 98.4 | 8.0 | 17.6 | 2.5 | 5768.0 | 3.8 | 228.5 | 0.0 | 100.0 | 94.9 | 6 |
| Real | h | 90.7 | Small farms | 11 | | 80.5 | | 7.4 | | 486.1 | | 59.5 | | 99.7 | |  |
| *G-RaFFe* |  | 90.0 |  | 130 | 6 | 89.5 | 17.5 | 8.7 | 2.5 | 884.7 | 40.8 | 97.1 | 42.4 | 100.0 | 98.8 | 6 |
| *Qrule* |  | 90.0 |  | 69 | 5 | 90.2 | 87.3 | 3.4 | 1.2 | 1063.3 | 76.9 | 120.4 | 46.9 | 100.0 | 99.8 | 3 |
| *Simmap* |  | 90.0 |  | 33 | 1 | 99.2 | 4.6 | 9.2 | 1.3 | 5850.9 | 40.7 | 751.8 | 0.0 | 100.0 | 96.7 | 6 |
