# Supplementary material for: Simple Process-Based Simulators for Generating Spatial Patterns of Habitat Loss and Fragmentation: A Review and Introduction to the G-RaFFe Model
Source: PLoS One. 2013 May 28;8(5):e64968. doi: 10.1371/journal.pone.0064968 (PMC3665680; doi:10.1371/journal.pone.0064968)
Supplement: Appendix S3 — Principal Component Analysis for the performance of all models in reproducing realistic spatial patterns. (DOCX) [file pone.0064968.s003.docx]

**Table S3:**

**Principal Component Analysis for the performance of all models in reproducing realistic spatial patterns.**

We present here the outcomes of PCA between real and simulated landscapes, based on each of the models *Simmap*, *Qrule*, Dinamica versions 1 to 4, and *G-RaFFe*. PCAs were individually performed on each habitat cover category, considering the Number of patches, Average patch size (Ha), Average Euclidean distance between patches (m), Landscape Shape Index (LSI), Fragment Shape Index (FSI), and Patch cohesion as grouping variables. Values represent the number of landscapes (N, either real or simulated) used in each analysis, and the proportion of total variance explained by the two first axes of the PCA.

| **Habitat cover category (%)** | **N Real Landscapes** | **N Simulated Landscapes** | **Variance explained Axis I** | **Variance explained Axis II** |
| --- | --- | --- | --- | --- |
| 5 | 2 | 39987 | 59 | 22 |
| 10 | 3 | 6242 | 64 | 20 |
| 15 | 2 | 4252 | 63 | 20 |
| 20 | 5 | 3971 | 61 | 19 |
| 25 | 5 | 2462 | 61 | 21 |
| 30 | 3 | 2077 | 62 | 20 |
| 35 | 4 | 2078 | 65 | 18 |
| 40 | 5 | 2078 | 67 | 19 |
| 45 | 2 | 2076 | 65 | 18 |
| 50 | 5 | 904 | 62 | 19 |
| 55 | 5 | 1500 | 64 | 16 |
| 60 | 1 | 891 | 58 | 17 |
| 65 | 2 | 1818 | 59 | 17 |
| 70 | 2 | 1236 | 57 | 16 |
| 75 | 1 | 387 | 57 | 17 |
| 80 | 2 | 286 | 57 | 19 |
| 85 | 0 | -- |  |  |
| 90 | 2 | 264 | 55 | 24 |
| **Total** | **51** | **72509 (x100)** |  |  |
